# Review of the millipede family Opisotretidae (Diplopoda, Polydesmida), with descriptions of new species

**DOI:** 10.3897/zookeys.302.5357

**Published:** 2013-05-20

**Authors:** Sergei I. Golovatch, Jean-Jacques Geoffroy, Pavel Stoev, Didier Vanden Spiegel

**Affiliations:** 1 Institute for Problems of Ecology and Evolution, Russian Academy of Sciences, Moscow, Russia; 2 Muséum national d’Histoire naturelle, Département Ecologie & Gestion de la Biodiversité, UMR 7204 CESCO, Brunoy, France; 3 National Museum of Natural History, Bulgarian Academy of Sciences, Sofia, Bulgaria; 4 Musée Royal de l’Afrique centrale, Tervuren, Belgium

**Keywords:** Diplopoda, Opisotretidae, taxonomy, new species, cave, China, Vietnam, Indonesia, Papua New Guinea

## Abstract

The small, basically Oriental family Opisotretidae is rediagnosed, reclassified, and shown to comprise the following seven genera, all keyed: *Carlotretus* Hoffman, 1980, with two species, including *Carlotretus triramus* **sp. n.** from southern China; *Corypholophus* Attems, 1938, with two species, one in Vietnam, the other in the Ryukyus, Japan; *Martensodesmus* Golovatch, 1987, with eight species, all keyed, including *Martensodesmus cattienensis* **sp. n.** from southern Vietnam, as well as *Martensodesmus bedosae* **sp. n.** and *Martensodesmus spiniger* **sp. n.** from southern China; *Opisotretus* Attems, 1907, with seven species, all keyed, including *Opisotretus beroni* **sp. n.** and *Opisotretus hagen* **sp. n.**, both from Papua New Guinea, *Opisotretus deharvengi* **sp. n.** from Sulawesi, Indonesia, and *Opisotretus spinosus* **sp. n.** from Nusakambangan Island, off Java, Indonesia; *Opisthoporodesmus* Silvestri, 1899, with six nominate species; *Retrodesmus* Chamberlin, 1945, with two species, i.e. the type-species *Retrodesmus dammermani* Chamberlin, 1945, from Java, Indonesia, revised from the holotype, and *Retrodesmus cavernicola* **sp. n.**, from Papua New Guinea; and *Solaenaulus* Attems, 1940, with two species. Comments are presented on the family’s possible relationships and palaeogeographic history. Instead of being considered as the sole component of the superfamily Opisotretoidea, the Opisotretidae is believed here to form one of the families of the diverse superfamily Trichopolydesmoidea, perhaps the sister-group to, if not immediately derived from, the pantropical family Fuhrmannodesmidae. The origin of Opisotretidae, previously dated as far back as the Triassic (220 Ma) in relation to the fragmentation of eastern Gondwanaland, mainly in the region of present-day Indonesia, could have had nothing to do with Gondwanaland. Opisotretids might have originated in mainland Southeast Asia well within the Cenozoic, with subsequent dispersals along the Himalayas in the West and across Indonesia (including New Guinea) in the East, also reaching as far north as the Ryukyus, Japan and Guangxi, southern China.

## Introduction

The small millipede family Opisotretidae was first proposed by Hoffman (1980) as a member of the superfamily Polydesmoidea, suborder Polydesmidea, to incorporate the following genera: *Opisotretus* Attems, 1907, *Corypholophus* Attems, 1938, *Solaenaulus* Attems, 1940 and *Carlotretus* Hoffman, 1980. Another two genera were also included, but only with qualifications: *Opisthoporodesmus* Silvestri, 1899 and *Retrodesmus* Chamberlin, 1945. Soon after that Hoffman (1982) firmly assigned all these six genera to Opisotretidae. Golovatch (1987) added one more genus, *Martensodesmus* Golovatch, 1987. Simonsen (1990), based on the results of a cladistic analysis, not only unequivocally included all these genera except *Carlotretus* (likely just forgotten) in Opisotretidae, but he also considered this family as the sole component of a separate superfamily, Opisotretoidea, in the infraorder Polydesmoides, suborder Polydesmidea.

Opisotretidae have hitherto been known to contain the following 20 species, arranged in alphabetic order:

1. *Carlotretus setosus* (Carl, 1922)

*Opisotretus setosus* Carl, 1922: 574.

*Solaenaulus setosus* – Attems 1940: 173.

*Carlotretus setosus* – Hoffman 1980: 188.

The type species of *Carlotretus* Hoffman, 1980, which Hoffman (1980) erected, based on certain gonopod traits alone. Originally described as *Opisotretus setosus* Carl, 1922, from Sumatra, Indonesia (Carl 1922).

2. *Corypholophus minutus* Attems, 1938

*Corypholophus minutus* Attems, 1938: 249.

*Corypholophus minutus* – Attems 1940: 190; Golovatch 1987: 205.

The type species of *Corypholophus*, originally described from near Nhatrang, southern Vietnam (Attems 1938), later recorded and clearly depicted from material taken from a locality in northern Vietnam (Golovatch 1987).

3. *Corypholophus ryukyuensis* Murakami, 1975

*Corypholophus ryukyuensis* Murakami, 1975: 108.

*Corypholophus ryukyuensis* – Golovatch 1987: 205; Nakamura and Korsós 2010: 82.

Described from several islands of the Ryukyu Archipelago, Japan (Murakami 1975), whence all later records have been summarized by Nakamura & Korsós (2010). Based on gonopod conformation, the assignment of *ryukyuensis* to *Corypholophus* was questioned by Golovatch (1987).

4. *Martensodesmus bicuspidatus* Golovatch, 1988

*Martensodesmus bicuspidatus* Golovatch, 1988: 32.

Described from Bhutan, Himalaya (Golovatch 1988).

5. *Martensodesmus excornis* Golovatch, 1988

*Martensodesmus excornis* Golovatch, 1988: 30.

Described from Bhutan, Himalaya (Golovatch 1988).

6. *Martensodesmus himalayensis* Golovatch, 1987

*Martensodesmus himalayensis* Golovatch, 1987: 205.

The type species of *Martensodesmus* Golovatch, 1987, described from Nepal, Himalaya (Golovatch 1987).

7. *Martensodesmus nagarjungicus* Golovatch, 1987

*Martensodesmus nagarjungicus* Golovatch, 1987: 207.

Described from Nepal, Himalaya (Golovatch 1987).

8. *Martensodesmus sherpa* Golovatch, 1987

*Martensodesmus sherpa* Golovatch, 1987: 206.

Described from Nepal, Himalaya (Golovatch 1987).

9. *Opisotretus euthus* Chamberlin, 1945

*Opisotretus euthus* Chamberlin, 1945: 4.

Rather poorly described from near Tjibodas (now Cibodas), Java, Indonesia (Chamberlin 1945).

10. *Opisotretus kraepelini* Attems, 1907

*Opisotretus kraepelini* Attems, 1907: 113.

*Opisotretus kraepelini* – Attems 1940: 150.

The type species of *Opisotretus* Attems, 1907, described from Mount Pangerango, Java, Indonesia (Attems 1907).

11. *Opisotretus mimus* Chamberlin, 1945

*Opisotretus mimus* Chamberlin, 1945: 4.

Very poorly described from ♀ material from near Tjibodas (now Cibodas), Java, Indonesia (Chamberlin 1945).

12. *Opisthoporodesmus anandrus* Chamberlin, 1945

*Opisthoporodesmus anandrus* Chamberlin, 1945: 3.

Very poorly described from a ♀ holotype from Doormanpad, Irian Jaya, central mountains, 1410–1450 m a.s.l., 3°24'S, 138°38'E, New Guinea, Papua Province, Indonesia (Chamberlin 1945).

13. *Opisthoporodesmus bacillifer* Carl, 1912

*Opisthoporodesmus bacillifer* Carl, 1912: 153.

*Opisthoporodesmus bacillifer* – Attems 1940: 152.

Very poorly described from two presumably subadult ♀♀ from Masarang, northern Sulawesi, Indonesia (Carl 1912).

14. *Opisthoporodesmus conservandus* Chamberlin, 1945

*Opisthoporodesmus conservandus* Chamberlin, 1945: 3.

Very poorly described from Prauwenbivak, Mamberamo River, 3°15'S, 138°35'E, ca 40 km SW of Sukarnapura, Irian Jaya, New Guinea, Papua Province, Indonesia (Chamberlin 1945).

15. *Opisthoporodesmus obtectus* Silvestri, 1899

*Opisthoporodesmus obtectus* Silvestri, 1899: 206.

*Opisthoporodesmus obtectus* – Attems 1940: 151.

The type species of *Opisthoporodesmus* Silvestri, 1899, describedfrom Tamara Island, near Berlinhafen (now Aitape), 3°13'S, 142°35'E, North Sepik Province, Papua New Guinea (Silvestri 1899).

16. *Opisthoporodesmus silvestri* Chamberlin, 1945

*Opisthoporodesmu s. silvestri* Chamberlin, 1945: 2.

Very poorly described from a ♀ holotype from Pionierbivak, 4°19'S, 141°55'E, Irian Jaya, New Guinea, Papua Province, Indonesia (Chamberlin 1945).

17. *Opisthoporodesmus simplex* Chamberlin, 1945

*Opisthoporodesmus simplex* Chamberlin, 1945: 4.

Very poorly described from ♀ and juvenile material from near Tjibodas (now Cibodas), Java, Indonesia (Chamberlin 1945).

18. *Retrodesmus dammermani* Chamberlin, 1945

*Retrodesmus dammermani* Chamberlin, 1945: 5.

The type species of *Retrodesmus* Chamberlin, 1945, quite poorly described from Tjibodas (now Cibodas), Java, Indonesia (Chamberlin 1945), briefly redescribed and properly illustrated below.

19. *Solaenaulus birmanicus* Carl, 1941

*Solaenaulus butteli*, ssp. *birmanica* Carl, 1941: 374.

*Solaenaulus butteli*– Jeekel 2006: 162.

*Solaenaulus birmanicus* – Golovatch et al. 2010: 143.

Originally described as *Solaenaulus butteli*, ssp. *birmanicus* (incorrectly spelled as “*birmanica*”), from Irawadi, Myanmar (Carl 1941). Golovatch et al. (2010) consider it a full species, as opposed to Jeekel (2006) who believed it was only a variety of the type species, thus deserving no taxonomic rank.

20. *Solaenaulus butteli* (Carl, 1922)

*Opisotretus butteli* Carl, 1922: 573.

*Solaenaulus butteli* – Attems 1940: 172; Carl 1941: 374; Jeekel 2006: 61; Golovatch et al. 2010: 140.

The type species of *Solaenaulus* Attems, 1940, which Attems (1940) erected, based on certain gonopod traits alone. Originally described as *Opisotretus butteli* Carl, 1922, from Sumatra, Indonesia (Carl 1922), since then recorded in Christmas Island, Indian Ocean, Australia (Jeekel 2006) and redescribed in due detail from material from Lae, Morobe Province, Papua New Guinea (Golovatch et al. 2010). Apparently, introduced to both latter localities.

In addition, unidentified Opisotretidae, provisionally referred to as ?*Corypholophus* sp. or *Martensodesmus* sp., respectively, occur also in Taiwan (Golovatch et al. 2011a) and southern Vietnam (Golovatch et al. 2011b).

As one can see from the above list, several species have been described too poorly to realistically become recognized. This holds especially true of what Chamberlin (1945) described in *Opisthoporodesmus*, making the compilation of even a superficial key to *Opisthoporodesmus* species impossible. The few he described from ♂ material must be revised, whereas the identities of the species which were based on ♀ and/or juvenile samples are bound to remain enigmatic until ♂ topotypes have been obtained and properly described. Since the main objective of the present paper is to address the generic classification of Opisotretidae in order to identify and name a number of fresh samples ranging from continental southern China, through Indochina and Indonesia, to Papua New Guinea, only *Retrodesmus dammermani*, fortunately an intact ♂ holotype, has been revised here.

### Abbreviations used

**AMNH** American Museum of Natural History, New York, U.S.A.

**IZAS** Institute of Zoology, Academia Sinica, Beijing, China

**MNHN** Muséum national d’Histoire naturelle, Paris, France

**MZB** Museum Zoologicum Bogoriense, Cibinong, Indonesia

**NMNHS** National Museum of Natural History, Sofia, Bulgaria

**SCAU** South China Agricultural University, Guangzhou, China

**SEM** Scanning electron microscopy

**ZMUC** National Museum of Natural History, Copenhagen, Denmark

**ZMUM** Zoological Museum, State University of Moscow, Moscow, Russia

## Material and methods

The bulk of the material treated below was taken by Louis Deharveng and Anne Bedos (MNHN) in Indonesia and China, as well as nearly entirely by Petar Beron (NMNHS) in Papua New Guinea. A few samples derive from ZMUM. The holotype of *Retrodesmus dammermani* was received on loan from AMNH. The holotypes from Indonesia have been housed in MZB, those from China in the collection of IZAS, whereas a few paratypes from China have been deposited in SCAU. Much of the material has been kept at MNHN, a few duplicates have also been donated to ZMUC and NMNHS, as indicated below.

SEM micrographs were taken using a JEOL JSM-6480LV scanning electron microscope.

After examination, SEM material was removed from stubs and returned to alcohol, all such samples from Papua New Guinea being kept in NMNHS, from the remaining places in MNHN.

### The main characters used in the classification of Opisotretidae

The following characters have been used for defining the genera in Opisotretidae, the only family in the entire order Polydesmida in which the gonopods are directed dorsolaterad, curving very strongly around coxae 8 along the sides of segment 7:

**Number of body segments.**

Like in most other families in Polydesmida, the number varies from 19 to 20, mostly being sex-characteristic. Thus, in *Carlotretus setosus*, *Corypholophus minutus* and *Opisotretus kraepelini*, the type species of their respective genera, the ♂ has 19 segments, whereas ♀♀ are unknown. Regrettably, Chamberlin (1945) did not care to mention the number of body segments in the ♀♀ of his *Opisotretus mimus* and *Opisotretus euthus*. *Opisthoporodesmus* species, perhaps including also *Opisthoporodesmus bacillifer* which was described from presumably subadult ♀♀ with 19 segments (Carl 1912), show equally 20 segments in both sexes. All of the remaining genera and species seem to have 19 segments in the ♂ versus 20 segments in the ♀.

**Number of rows of setae on body metaterga.**

Only two known species show two transverse rows on the metaterga: *Carlotretus setosus* and *Corypholophus ryukyuensis*, as opposed to the other species which clearly have three transverse rows of bacilliform setae, these sometimes being evidently shifted caudad. However, this character appears to be only species-specific, as one of the new species of *Martensodesmus* described below also has only two rows of tergal setae.

These setae are longitudinally ribbed (Figs 5F, 14I, 19L, 22J, 24J, 28B, 30L, 34B, C, 37J, 40B). However, similar setae occur in certain Fuhrmannodesmidae as well. For example, a still unpublished fuhrmannodesmid from Vietnam shows tergal setae of two types, one claviform (Fig. 42M), the other bacilliform (Fig. 42F), both ribbed the same way. Moreover, some, but not all, species of the genus *Boreviulisoma* Brolemann, 1928, representing the distantly related family Paradoxosomatidae, also have similarly ribbed bacilliform setae (Reboleira and Enghoff 2013).

**Metatergal sculpture.**

The pattern of metatergal sculture is that typical of the Polydesmidea, i.e. three transverse rows of polygonal bosses, with a more or less deep sulcus separating the first row from the two following ones. Each boss is typically surmounted by a seta sometimes borne on a small knob, the pattern being 3+3 per row (see above). In Opisotretidae, only few species show very distinct bosses, like those observed in *Solaenaulus butteli* (Figs 4, 5) or *Opisotretus beroni* sp. n. (Figs 17A–F), whereas in most species the bosses tend to be poorly visible to virtually untraceable (Figs 24A–F), whereas the transverse sulcus is largely superficial.

**Location of ozopores.**

**Figure 1.**
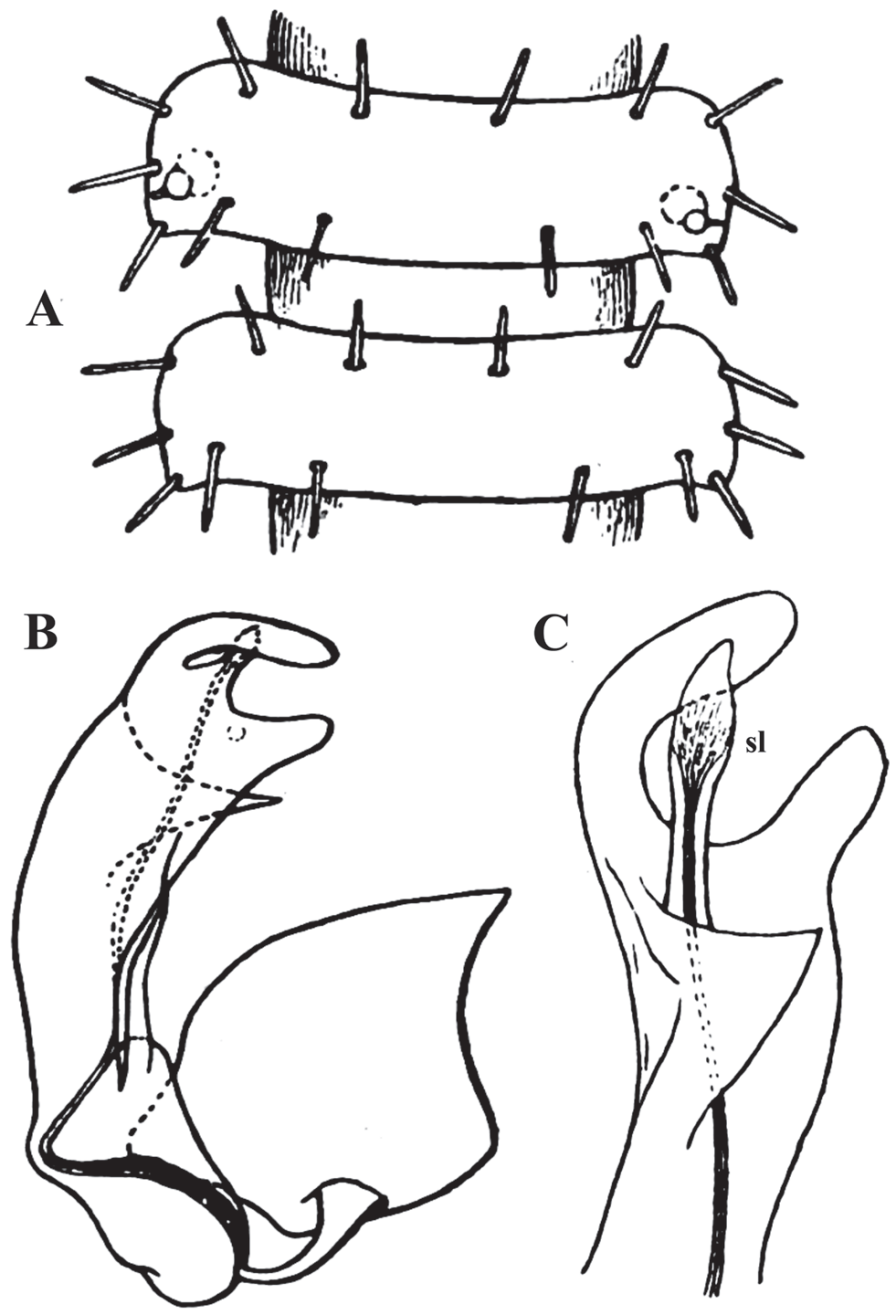
*Carlotretus setosus* Carl, 1922; ♂ holotype from Sumatra, Indonesia; **A** midbody segments, dorsal view **B, C** right gonopod and its apical part, mesal and lateral views, respectively. Depicted not to scale. After Carl (1922).

The location of ozopores is often quite peculiar in species of Opisotretidae. The pore formula always being normal, 5, 7, 9, 10, 12, 13, 15-18 (19), the ozopores are normally placed near the caudolateral corner of paraterga, very to quite close to the caudal margin of the tergite (Figs 1A, 5B, G, 8A, B, 11A, B, 14B, G, K, 17C, L, 22C, E, M, 24B, C, K, 26B, C, 30B, C, E, F, K, 31B, 33B, C, F, 34B, 36, 37B, E, F, 39B–E, 40B). Chamberlin (1945) paid special attention to this character when assigning his species to either *Opisotretus* or *Opisthoporodesmus*, or *Retrodesmus*. In particular, he tended to treat all species with ozopores placed especially close to the caudal metatergal margin in *Opisotretus*, apparently following Attems (1907), whereas the species with the ozopores slightly more strongly removed forward from the caudal margin he placed in *Opisthoporodesmus*. In the type species of *Retrodesmus*, the ozopore is well removed from the caudal corner of paraterga, lying closer to their lateral margin, but in a new congener described below the ozopores lie just at the caudal margin, this being more typical of the family.

However, this feature must be admitted as not being unique to and characteristic of some Opisotretidae alone. Thus, many species of Fuhrmannodesmidae possess ozopores which also flush open at the caudolateral corner of poriferous paraterga quite close to very close to the caudal margin, in South America (Golovatch 1994) and Vietnam (Figs 42C, F). In general, this condition strongly depends on the degree of development of paraterga which varies between species, as well as between segments. The more strongly the caudal corner of a paratergum is drawn caudad, this being increasingly marked towards the telson, the closer the ozopore to the caudal margin.

In other words, the distinctions based on ozopore location are species-specific at most and clearly fail to characterize opisotretid genera.

**Shape of paraterga.**

Variation in the degree of development of paraterga is great, ranging from very poorly developed, e.g. in *Solaenaulus butteli* (Figs 4, 5), to very broad and upturned, e.g. in *Retrodesmus cavernicola* sp. n. (Figs 14A–E, G, H). Paraterga tend to be more strongly developed, up to directed dorsolaterad, only in *Opisthoporodesmus* and *Retrodesmus* species, where the midbody paraterga usually show considerable shoulders anterolaterally and a no less considerable emargination caudally (Figs 11A, B, 12). In the remaining Opisotretidae, however, the paraterga tend to be modest to very modest (Figs 1A, 3E, 8A–C, 33A–F, H, 34A, 37A–F, 39A–E, H, J), especially in ♀♀ and juveniles.

The presence of especially prominent shoulders seems to correlate positively with a shift caudad of the transverse rows of tergal setae. In such species, the frontal row of setae is situated close to the metatergite’s midway sulcus, whereas both following rows are considerably shortened in extent, strongly shifted to the caudal margin of the tergite and placed very close to each other (Figs 11A, B).

♂**head modifications.**

**Figure 2.**
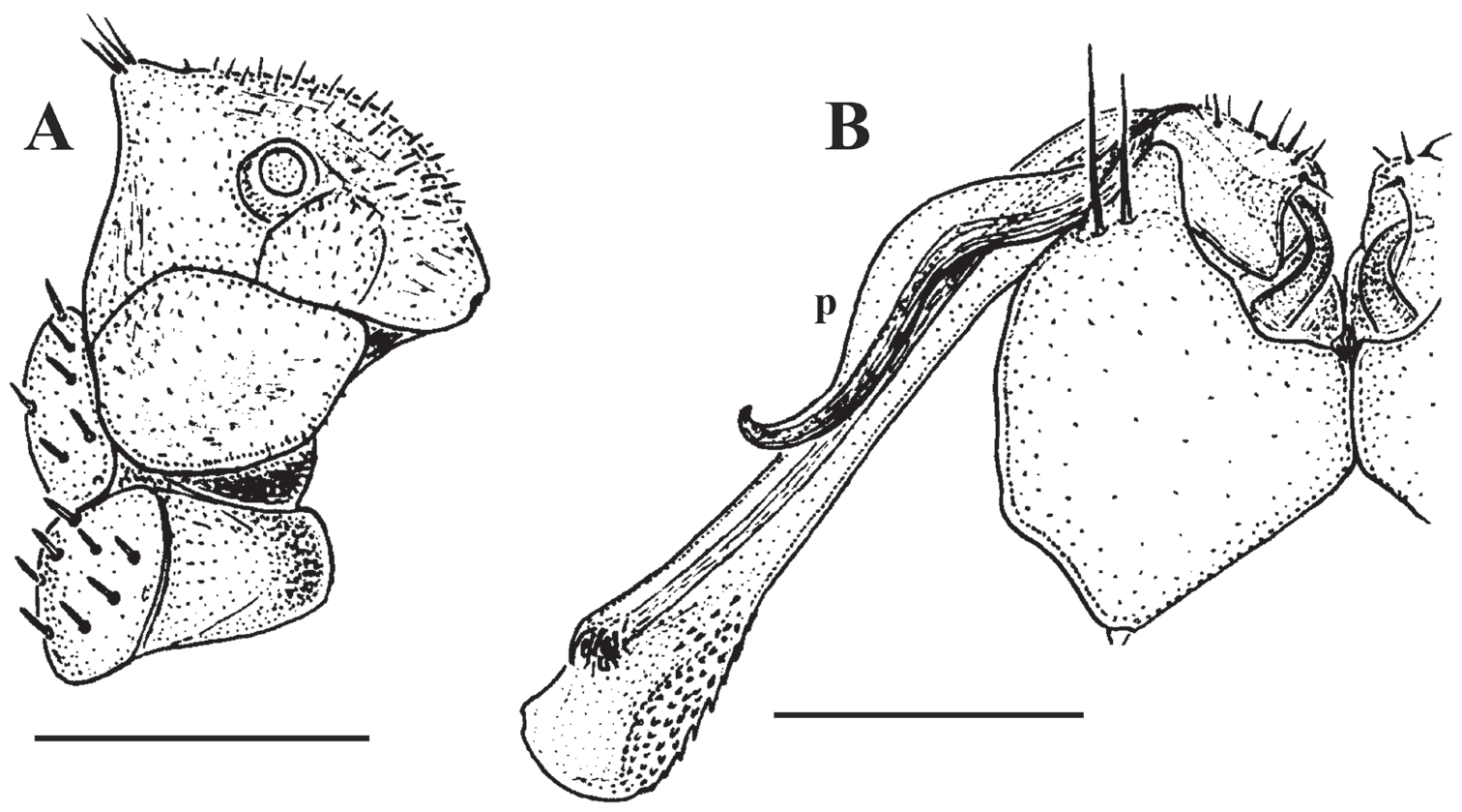
*Corypholophus minutus* Attems, 1938, ♂ from left bank of Ma River, Von Mai, Mai Tiao Distr., Hoa Binh Prov., northern Vietnam; **A** head, lateral view **B** left gonopod, caudal view. – Scale bars: **A** 0.3 mm; **B** 0.1 mm. After Golovatch (1987).

The ♂ vertex of several Opisotretidae is modified. In particular, *Martensodesmus*, among other things, was first distinguished by ♂ vertigial modifications usually traceable as humps or tubercles above the antennal sockets (Golovatch 1987). Later this feature had to be abandoned as a generic-level character after the discovery of *Martensodesmus excornis* which lacks any such modifications. Moreover, sexual dimorphism in *Martensodesmus bicuspidatus* was found to concern not only a complex structure (a fossa with two cusps of filaments) on the ♂ vertex, but also the shape of the collum (Golovatch 1988). ♂ vertigial modifications are also known in *Corypholophus minutus* (a hump with a tuft, Fig. 2A) and two new *Opisotretus* described below.

**Figure 3.**
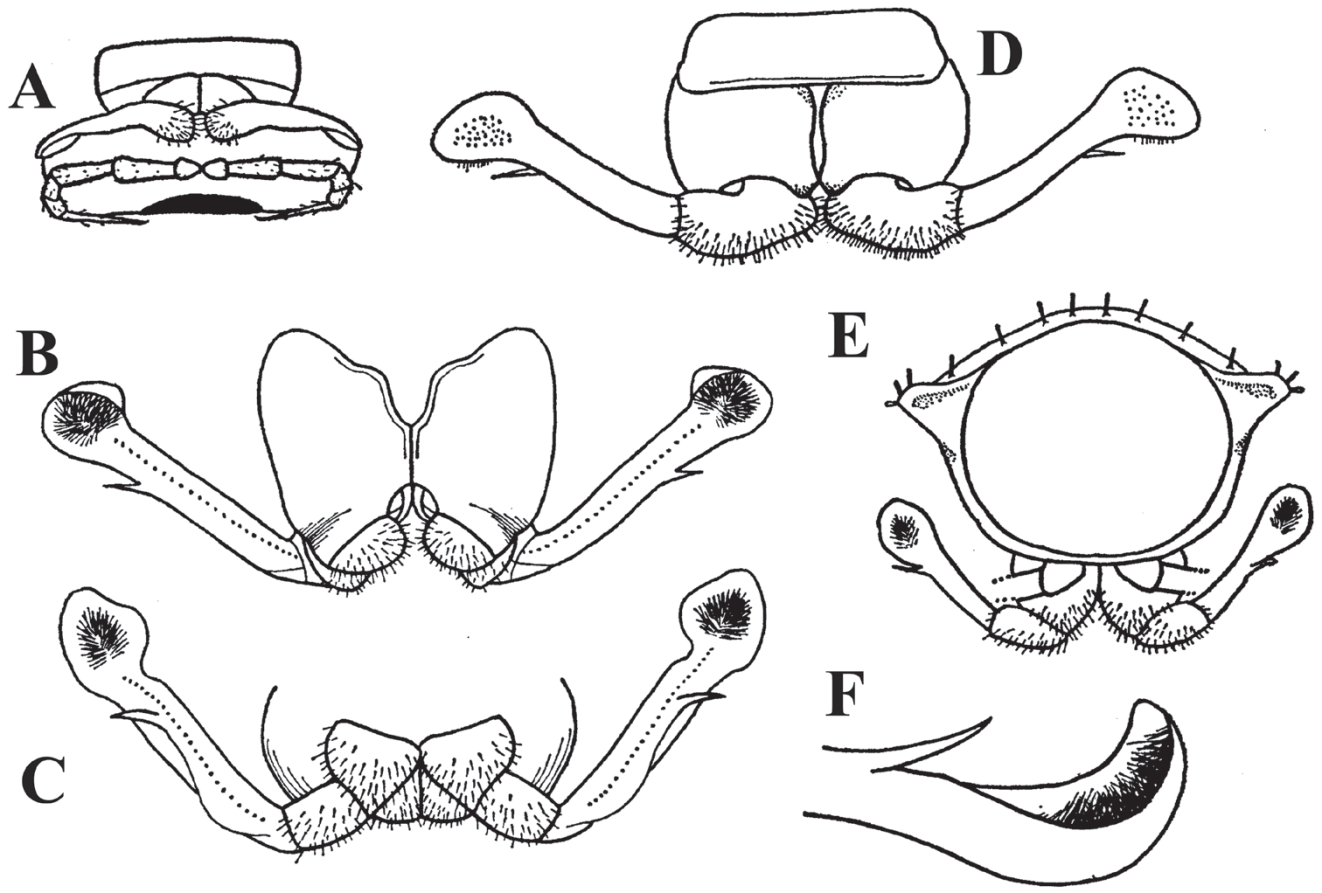
*Corypholophus ryukyuensis* Murakami, 1975, ♂ paratype from Ryukyu Islands, Japan; **A–E** both gonopods *in situ*, ventral, caudal, subcaudal and caudal views, respectively **F** gonopod tip, dorsal view. Depicted not to scale. After Murakami (1975).

**Figure 4.**
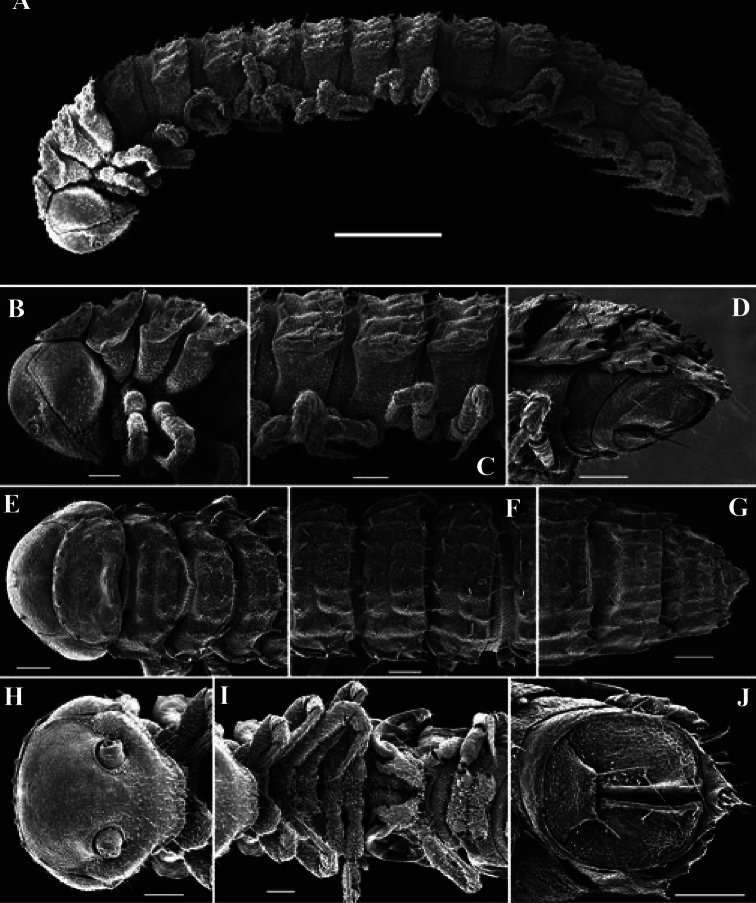
*Solaenaulus butteli* (Carl, 1922), ♂ from Lae, Papua New Guinea; **A** habitus, lateral view **B, E, H** anterior body part, lateral, dorsal and ventral views, respectively **C, F, I** midbody segments, lateral, dorsal and ventral views, respectively **D, G, J** posterior body part, lateral, dorsal and ventral views, respectively. – Scale bars: **A** 0.5 mm; **B–I** 0.1 mm; J, 0.12 mm. After Golovatch et al. (2010).

However, like in the case of ozopore location (see above), similar vertigial modifications in the ♂ concern numerous species of Fuhrmannodesmidae, including those occurring in South America (Golovatch 1994) and Vietnam (Figs 42D, G).

**Legs.**

**Figure 5.**
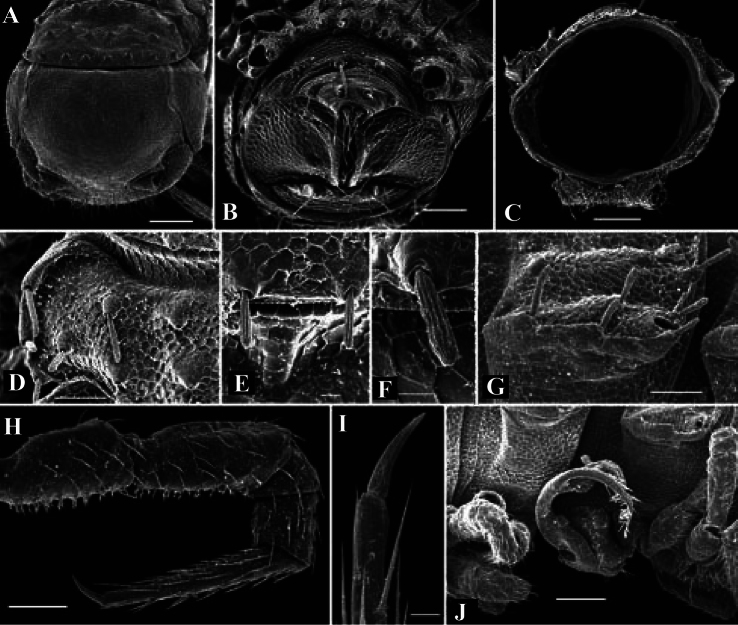
*Solaenaulus butteli* (Carl, 1922), ♂ from Lae, Papua New Guinea **A** head and collum, dorsal view **B** metatergum 18 and telson, caudal view **C** cross-section of a midbody segment, caudal view **D–G** tegument texture and tergal setae, dorsal, dorsal, dorsal and lateral views, respectively **H** midbody leg **I** claw **J** segment 7 with left gonopod *in situ*. – Scale bars: **A, C, J** 0.1 mm; **B, D, G, H** 0.05 mm; **E** 0.02 mm; **F, I** 0.01 mm. After Golovatch et al. (2010).

Variation in leg length and armament in Opisotretidae is pronounced, ranging from short and stout, sometimes also supplied with special ventral trichomes in the ♂, e.g. in *Solaenaulus butteli* (Fig. 5H), to extremely long and slender, e.g. in *Retrodesmus cavernicola* sp. n. (Figs 15B, 16A), but most species show medium-sized, moderately stout legs which are usually devoid of special trichomes in the ♂ and thus fail to differ much between the sexes. Claw length seems to vary proportionately to leg length (Figs 5H, I, 8C, 15B, 16A, 27K, 28D).

**Gonopod structure.**

As usual in the systematics of any subgroup of Polydesmida, the gonopods offer most of the characters deemed useful, if not crucial, for the discrimination of genera and species. This fully applies to Opisotretidae as well.

**Figure 6.**
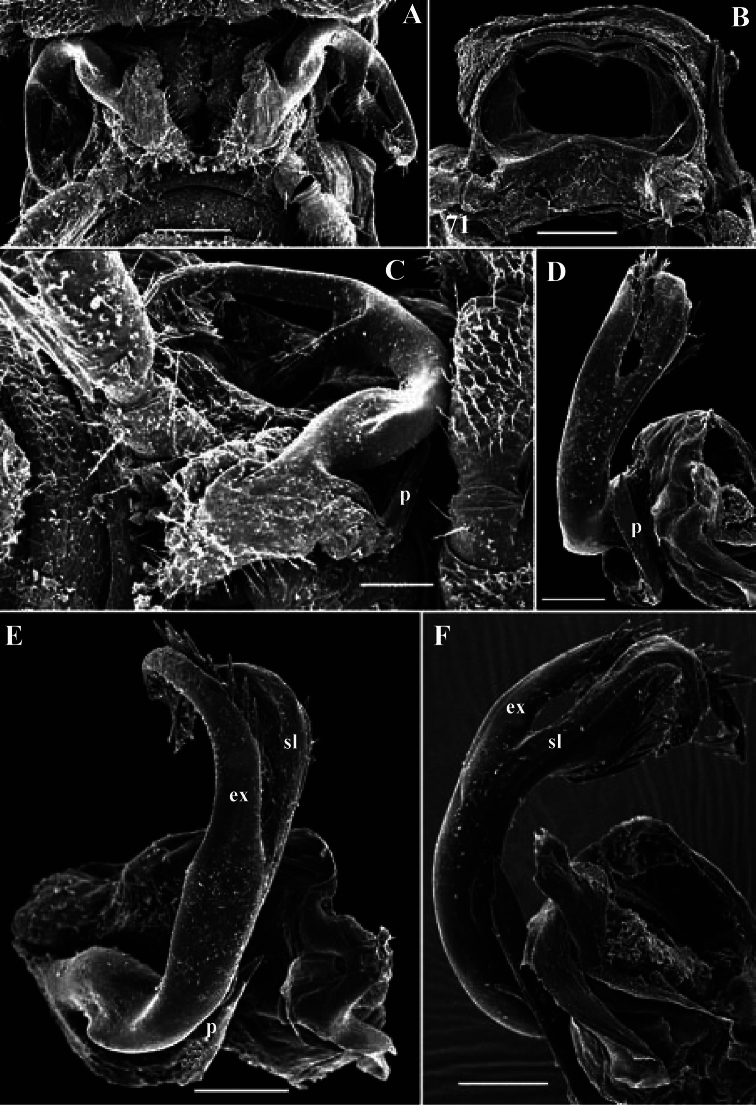
*Solaenaulus butteli* (Carl, 1922), ♂ from Lae, Papua New Guinea **A** both gonopods *in situ*, ventral view **B** gonopod aperture, ventral view **C–F** individual gonopods, subventral, subfrontal, lateral and mesal views, respectively. – Scale bars: **A, B** 0.05 mm; **C–F** 0.01 mm. After Golovatch et al. (2010).

As noted above, the gonopods in Opisotretidae are really unique in obviously having rather small, subglobose, medially fused and nearly fully exposed coxae, these being only very poorly sunken into an unusually small gonocoel. The gonopod aperture is invariably obcordate (Fig. 6B). The coxae support the usual cannulae medially and elongated, sometimes strongly curved telopodites laterally. The telopodites are directed dorsolaterad, curving, often very strongly, around coxae 8 along the sides of segment 7. The seminal groove runs along most of the telopodite’s extent to terminate distally either on a special branch or tooth (= solenomere), or flush open on the surface, or debauch inside an accessory seminal chamber which normally is supplied with a hairy pulvillus.

**Figure 7.**
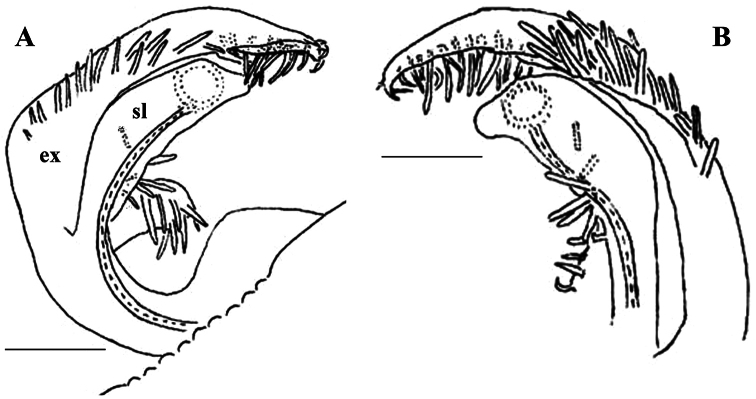
*Solaenaulus butteli* (Carl, 1922), ♂ from Lae, Papua New Guinea; **A, B** left gonopod, lateral and submesal views, respectively. – Scale bars: A, 0.4 mm; B, 0.1 mm. After Golovatch et al. (2010).

Against this general pattern, various species and genera show several important modifications. Species in two of the genera are recognized for the presence of a very peculiar frontobasal process placed on the ventral side of the femorite: in *Corypholophus minutus*, this process (**p**) is neatly attached to a rather slender, unipartite and suberect gonotelopodite, and it carries an additional groove (Fig. 2B), whereas **p** in *Solaenaulus* is devoid of a groove, being well separated from a strongly unciform, bipartite telopodite beset with bacilliform structures distally (Figs 6A, C–F, 7). Because *Corypholophus ryukyuensis* has no process **p** (Fig. 3), its generic assignment has been questioned (Golovatch 1987). However, since a well-developed **p** also occurs in a new species of *Martensodesmus* described below, this character must be regarded as being only species-specific. This clearly supports maintaining *Corypholophus ryukyuensis* in *Corypholophus*, even though *Corypholophus minutus* has a **p** (Fig. 2B).

**Figure 8.**
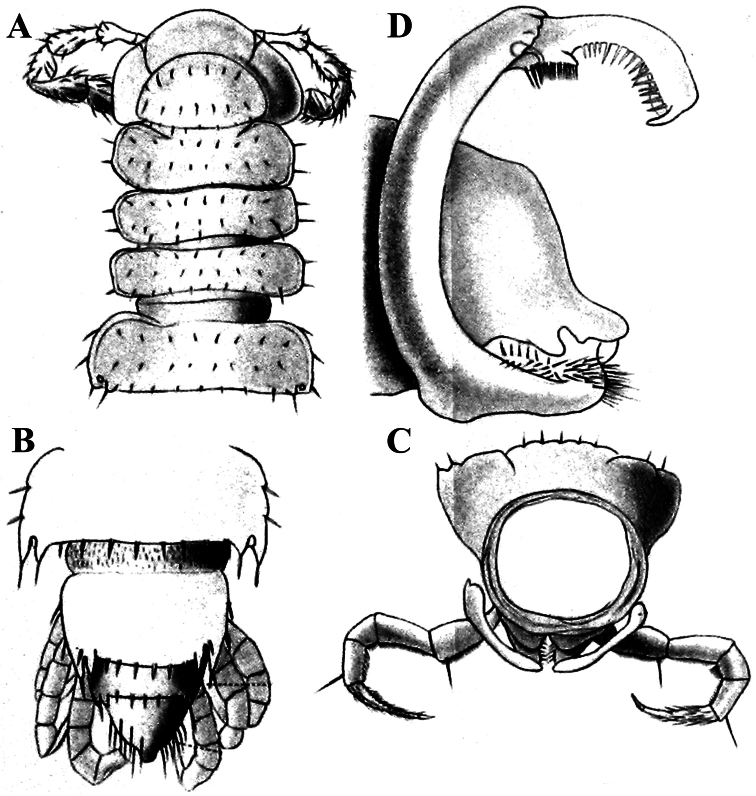
*Opisotretus kraepelini* Attems, 1907, ♂ holotype from Java, Indonesia **A, B** anterior and posterior body parts, respectively **C** cross-section of segment 7, frontal view **D** left gonopod, sublateral view. Depicted not to scale. After Attems (1907).

*Carlotretus* seems to be the only genus in Opisotretidae in which the distal part of a unipartite gonotelopodite is totally free from fringes or bacilli, including a long, erect and simple solenomere (**sl**) (Figs 1B, C, 40D–H, 41). *Solaenaulus*, in addition to **p**, also shows a prominent solenomere (**sl**) attached closely to a similarly long exomere (**ex**), both the branches being curved and abundantly ornamented with bacilli (Figs 6, 7). In all other opisotretid genera and species, the solenomere is a rather small denticle or lobule at most. The gonopods of *Opisotretus*, of *Opisotretus kraepelini* at least (Fig. 8D), look very similar to those of *Solaenaulus*, especially as regards the unciform appearance and the distal ornamentation of the telopodite, but the latter in *Opisotretus* is unipartite, sometimes being also devoid even of a vestigial solenomere (Figs 26F, G). *Opisthoporodesmus*, at least *Opisthoporodesmus obtectus*, *Martensodesmus* and *Retrodesmus* share the gonopod telopodite being rather short, poorly curved and, in the former two genera, modestly ornamented distally. The gonotelopodite in *Martensodesmus* species is often more or less hollow or flattened on the caudal face and carries considerable lobes or processes, sometimes including **p**. In *Opisthoporodesmus obtectus*, the gonopod telopodite (Fig. 11C) is very simple, attenuating distad and virtually fully devoid of a trichome other than the one on a subterminal hairy pulvillus. In contrast, *Retrodesmus* has an enlarged, bifid and elaborate tip of the gonotelopodite, one of its apical branches being beset with bacilliform ornamentations, but showing neither a solenomere nor an accessory seminal chamber, nor a hairy pulvillus.

**Vulva.**

No special studies have been conducted on the conformation of the vulva in Opisotretidae. Only Chamberlin (1945) depicted the vulva of *Opisthoporodesmus silvestri* as showing a remarkable subelliptic lobe. The epigynal crest has never been described either. Because these structures are too small and inconspicuous in the samples we have examined, they have been omitted from the descriptions.

**Figure 9.**
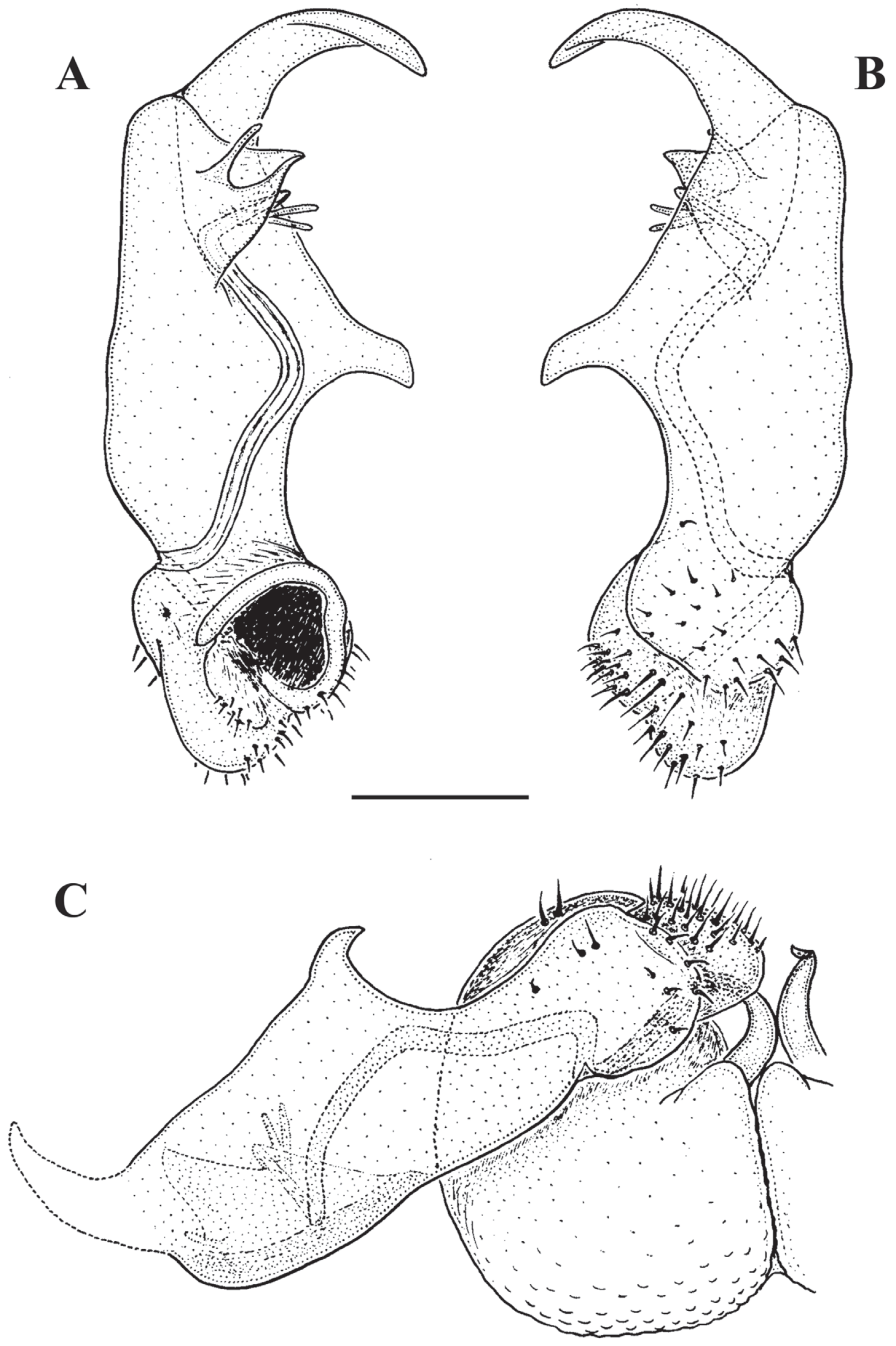
*Martensodesmus himalayensis* Golovatch, 1987, ♂ paratype from Nepal; **A, B** right gonopod, subcaudal and subfrontal views, respectively **C** left gonopod, frontal view. – Scale bar: 0.1 mm. After Golovatch (1987).

**Figure 10.**
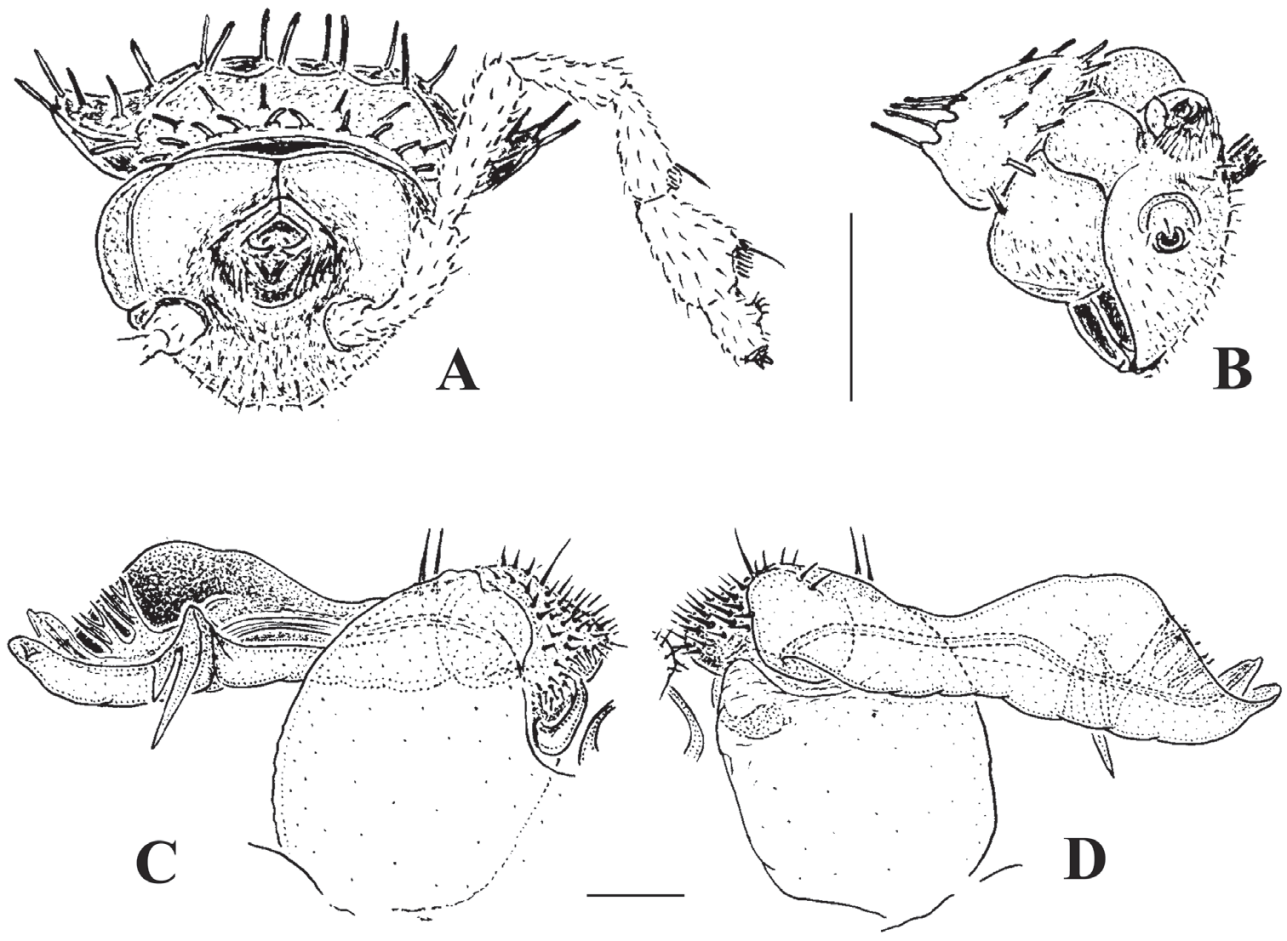
*Martensodesmus bicuspidatus* Golovatch, 1988, ♂ paratype from Bhutan; **A, B** head and collum, frontal and lateral views, respectively **C, D** right gonopod, caudal and frontal views, respectively. – Scale bars: **A, B** 0.5 mm; **C, D** 0.1 mm. After Golovatch (1988).

**Figure 11.**
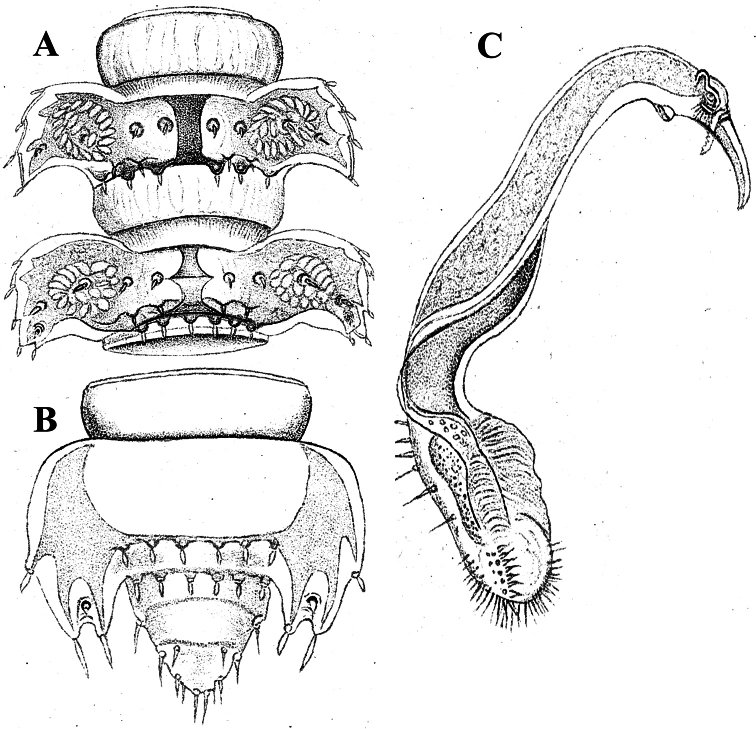
*Opisthoporodesmus obtectus* Silvestri, 1899, ♂ ?holotype from Papua New Guinea; **A** segments 8 and 9, dorsal view **B** posterior body part, dorsal view **C** right gonopod, subcaudal view. Depicted not to scale. After Silvestri (1899).

## Generic reclassification

Based on the above information, as well as facing the need to properly allocate several new species described below, we propose the following new classification of Opisotretidae.

### Family Opisotretidae

Hoffman, 1980

http://species-id.net/wiki/Opisotretidae

Opisotretidae Hoffman, 1980: 176, 188.
Opisotretidae – Hoffman 1982: 722; Simonsen 1990: 53, 82.

#### Diagnosis.

A family of the suborder Polydesmidea with 19 (♂) or 20 (♂, ♀) segments. Body small to very small (3–16 mm long). Tegument microalveolate, limbus microspiculate. ♂ head with or without vertigial modifications. Antennae geniculate between segments 5 and 6, antennomeres 5 and 6 each with a compact group of bacilliform sensilla distodorsally. Metaterga with 2 or 3 regular, transverse rows of bacilliform, longitudinally ribbed setae sometimes borne on minute knobs; frontal margin of midbody paraterga only seldom forming clear-cut shoulders; side margin of paraterga often slightly incised, with 2 or 3 bacilliform setae. Pore formula normal, ozopores flush open dorsally, usually near to very near caudolateral corner of paraterga, only seldom clearly removed from caudal margin. Legs rather short to long, ♂ ones often stouter and longer, sometimes with peculiar, bi- or trifid ventral trichomes, but sphaerotrichomes missing.

Gonopods peculiar in having rather small, subglobose, medially fused coxae nearly fully exposed in a small gonocoel; coxae at most only slightly setose ventrally, supporting usual cannulae medially and elongated, sometimes strongly curved telopodites laterally; the latter directed dorsolaterad, curving, often very strongly, around coxae 8 along sides of segment 7; seminal groove running along most of telopodite on caudal face to terminate distally either on a special branch or tooth (= solenomere), or flush open on caudal surface, or debauching inside an accessory seminal chamber which normally, but not always, is supplied with a hairy pulvillus.

#### Type genus.

*Opisotretus* Attems, 1907.

#### Remarks.

The above somatic features of Opisotretidae are in no way unique to the family, at least sometimes being also encountered, in various combinations, in the other families of the micropolydesmoid superfamily Trichopolydesmoidea, such as Fuhrmannodesmidae, Trichopolydesmidae, Macrosternodesmidae, Mastigonodesmidae and Nearctodesmidae (e.g. Golovatch 1994, 2011). This can also be said about the basically finely microspiculate limbus obviously characteristic of most of the Polydesmidea. It is only the gonopod structure that is truly characteristic of Opisotretidae, the family formally representing a superfamily of its own, the Opisotretoidea (Simonsen 1990). Superficially, female and/or juvenile Opisotretidae are not or only barely distinguishable from the often sympatric or even syntopic female or juvenile Fuhrmannodesmidae, the latter family dominating most of the tropical micropolydesmoid faunas. In this connection, we rather believe that Opisotretidae is also a family of Trichopolydesmoidea, probably the closest to Fuhrmannodesmidae (see below).

### Genus Carlotretus

Hoffman, 1980

http://species-id.net/wiki/Carlotretus

Carlotretus Hoffman, 1980: 176, 188.
Carlotretus – Hoffman 1982: 722.

#### Diagnosis.

A genus of Opisotretidae with 19 (♂) or 20 (♀) body segments. ♂ head without modifications. Metaterga with two regular, transverse rows of bacilliform setae. Frontolateral margin of midbody paraterga devoid of evident shoulders. Ozopore lying close to caudal margin of paratergite’s caudolateral corner.

Gonopod telopodite rather stout, unipartite, at best slightly hollow on caudal face; distal part devoid of ornamentations (spines or setae), being a long and simple solenomere (**sl**) supplied with lobes or processes either subtending it or lying at its base. Neither an accessory seminal chamber nor a hairy pulvillus (Figs 1B, C, 38B–E, 40D–H, 41).

#### Type species.

*Opisotretus setosus* Carl, 1922, by original designation of Hoffman (1980).

#### Remarks.

In addition to the type species, this genus includes a new congener described below. The differences are depicted in Fig. 1 and Figs 38, 40, 41, being also mentioned in the diagnosis of *Carlotretus triramus* sp. n.

### Genus Corypholophus

Attems, 1938

http://species-id.net/wiki/Corypholophus

Corypholophus Attems, 1938: 249.
Corypholophus – Attems 1940: 190; Murakami 1975: 108; Hoffman 1980: 176, 188; 1982: 722; Golovatch 1987: 205; Simonsen 1990: 53.

#### Diagnosis.

A genus of Opisotretidae with 19 (♂) or 20 (♀) body segments. ♂ vertex with or without modifications. Metaterga with 2 or 3 regular, transverse rows of bacilliform setae. Frontolateral margin of midbody paraterga devoid of shoulders. Ozopore lying close to caudal margin of paratergite’s caudolateral corner.

Gonopod telopodite slender, unipartite, slightly hollow on caudal face only distally; basal frontoventral process (**p**) either present or absent; distal part devoid of ornamentations (spines, bacilli or setae), lobes or prominent processes, at most microdenticulate near both a small accessory seminal chamber and a hairy pulvillus (Figs 2B, 3).

**Type species:** *Corypholophus minutus* Attems, 1938, by original designation.

**Remarks.** This genus also includes *Corypholophus ryukyuensis* from the Ryukyus, Japan (and Taiwan?). The differences between these two species are depicted in Fig. 2B and Fig. 3.

### Genus Martensodesmus

Golovatch, 1987

http://species-id.net/wiki/Martensodesmus

Martensodesmus Golovatch, 1987: 203.
Martensodesmus – Golovatch 1988: 34; Simonsen 1990: 53.

#### Diagnosis.

A genus of Opisotretidae with 19 (♂) or 20 (♀) body segments. ♂ head often, but not always, with modifications on vertex, collum rarely enlarged. Most of metaterga with 2 (more rarely) or 3 (more usually) regular, transverse rows of bacilliform setae. Frontolateral margin of midbody paraterga usually without evident shoulders, in any event not so strongly developed as to cause a caudad shift of the rows of tergal setae. Ozopore lying from close to, to rather far in front of caudal margin of paratergite’s caudolateral corner.

Gonopod telopodite rather stout, at least basal half so, unipartite, usually only faintly curved, slightly, more usually clearly, hollow/excavate/flattened on caudal face all along; parabasal and/or distal parts with lobes or processes, sometimes including **p**; both accessory seminal chamber and hairy pulvillus wanting, but a very short, dentiform solenomere usually ornamented with a few bacilli- or setiform structures nearby often present (Figs 9, 10C, D, 28E–I, 29, 31E–H, 32, 35).

#### Type species.

*Martensodesmus himalayensis* Golovatch, 1987, by original designation.

**Remarks.** In addition to the type species, the genus currently contains further four Himalayan congeners: *Martensodesmus bicuspidatus* Golovatch, 1988, *Martensodesmus excornis* Golovatch, 1988, *Martensodesmus nagarjungicus* Golovatch, 1987 and *Martensodesmus sherpa* Golovatch, 1987, as well as one new species in Vietnam and two more in southern China. A key to *Martensodesmus* species is given below.

### Genus Opisotretus

Attems, 1907

http://species-id.net/wiki/Opisotretus

Opisotretus Attems, 1907: 113.
Opisotretus – Attems 1940: 150; Chamberlin 1945: 4; Hoffman 1980: 176, 188; 1982: 722; Simonsen 1990: 53.

#### Diagnosis.

A genus of Opisotretidae with 19 (♂) or 20 (♀) body segments. ♂ vertex with or without modifications. Metaterga with three regular, transverse rows of bacilliform setae. Frontal margin of midbody paraterga devoid of obvious shoulders. Ozopore usually lying close to very close to caudal margin of paratergite’s caudolateral corner.

Gonopod telopodite elongate, unciform, unipartite; distal part beset with ornamentations (small spines, bacilli or setae) and at least with one evident process, either devoid of or supplied with a short solenomere, but with both an evident accessory seminal chamber and a hairy pulvillus (Figs 8D, 18B–D, 20C, D, 21, 23C, D, 25, 26F, G).

#### Type species.

*Opisotretus kraepelini* Attems 1907, by monotypy.

#### Remarks.

In addition to the type species, the genus currently contains two described congeners: *Opisotretus euthus* Chamberlin, 1945 and *Opisotretus mimus* Chamberlin, 1945. Because the gonopods of *Opisotretus euthus* are indeed very similar to those of *Opisotretus kraepelini* as depicted by Chamberlin (1945), the former species is definitely congeneric with the latter one. The identity of *Opisotretus mimus*, however, remains uncertain, but superficially it strongly reminds of *Peronorchus parvicollis* Attems, 1907, a species we think belongs in the family Fuhrmannodesmidae. It was originally described from Buitenzorg (= Bogor), Java, Indonesia  (Attems 1907) and seems to be very similar to an opisotretid in showing long bacilliform tergal setae arranged in three transverse rows, notably reduced paraterga, and the ozopores located near the paratergite’s caudal corner. Interestingly, Mauriès and Geoffroy (1999), when redescribing *Peronorchus parvicollis* from material taken on Mauritius, Indian Ocean, assigned this genus to the family Trichopolydesmidae, as opposed to Hoffman (1980) who had left *Peronorchus* among the genera of Polydesmidea of uncertain status and family position.

Four new species described below also belong in *Opisotretus*. A key to all seven *Opisotretus* species, including *Opisotretus mimus*, is given below.

### Genus Opisthoporodesmus

Silvestri, 1899

http://species-id.net/wiki/Opisthoporodesmus

Opisthoporodesmus Silvestri, 1899: 206.
Opisthoporodesmus – Attems 1940: 151; Chamberlin 1945: 2-4; Hoffman 1982: 722; Simonsen 1990: 53.

#### Diagnosis.

A genus of Opisotretidae with 20 body segments (♂, ♀). ♂ vertex without modifications. Metaterga with three regular, transverse rows of bacilliform setae, but, probably in conjunction with frontolateral margin of midbody paraterga bearing prominent shoulders, at least sometimes all three rows strongly shifted caudad, last two being also abbreviated. Ozopore usually lying close to caudal margin of paratergite’s caudolateral corner.

Gonopod telopodite elongate, subunciform, unipartite, markedly attenuating distad; distal part with only a few small outgrowths at best, devoid of both bacilliform ornamentations and a solenomere, but supplied with both a small accessory seminal chamber and a hairy pulvillus (Fig. 11C).

#### Type species.

*Opisthoporodesmus obtectus* Silvestri, 1899, by monotypy.

#### Remarks.

In addition to the type species, the genus currently contains five formal congeners: *Opisthoporodesmus anandrus* Chamberlin, 1945, *Opisthoporodesmus bacillifer* Carl, 1912, *Opisthoporodesmus conservandus* Chamberlin, 1945, *Opisthoporodesmus silvestri* Chamberlin, 1945 and *Opisthoporodesmus simplex* Chamberlin, 1945. As these five species require revision and their identities remain uncertain, no key to *Opisthoporodesmus* species is possible for the time being.

### Genus Retrodesmus

Chamberlin, 1945

http://species-id.net/wiki/Retrodesmus

Retrodesmus Chamberlin, 1945: 4.
Retrodesmus – Hoffman 1982: 722; Simonsen 1990: 53.

#### Diagnosis.

A genus of Opisotretidae with 19 (♂) or 20 (♀) body segments. ♂ vertex without modifications. Metaterga with three regular, transverse rows of bacilliform setae, but, in conjunction with frontolateral margin of midbody paraterga bearing evident shoulders, all three rows strongly shifted caudad, last two being also abbreviated. Ozopore from well removed from, to very near caudal margin of paratergite’s caudolateral incision.

Gonopod telopodite rather stout, only slightly curved, unipartite, divided only distally into a frontal stump heavily beset with bacilliform ornamentions and a simple to complex caudal branch; seminal groove terminating near base of both these branches; neither a solenomere nor a hairy pulvillus (Figs 13B, 15C, D, 16B, C), only sometimes with a visible accessory seminal chamber.

#### Type species.

*Retrodesmus dammermani* Chamberlin, 1945, by original designation.

#### Remarks.

**Figure 12.**
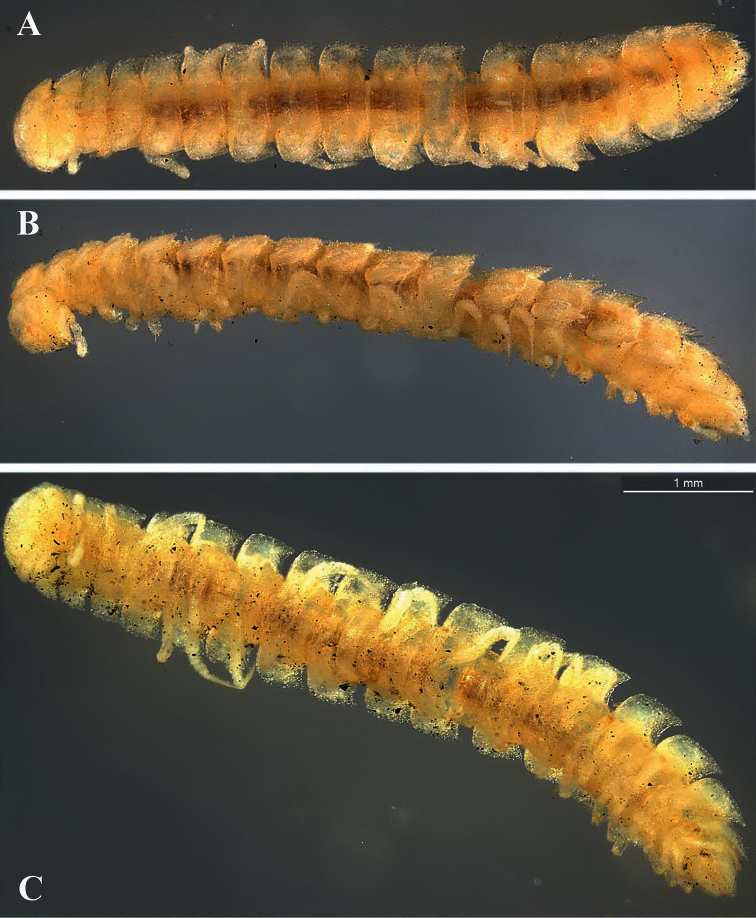
*Retrodesmus dammermani* Chamberlin, 1945, ♂ holotype from Java, Indonesia; **A–C** habitus, dorsal, lateral and ventral views, respectively.

**Figure 13.**
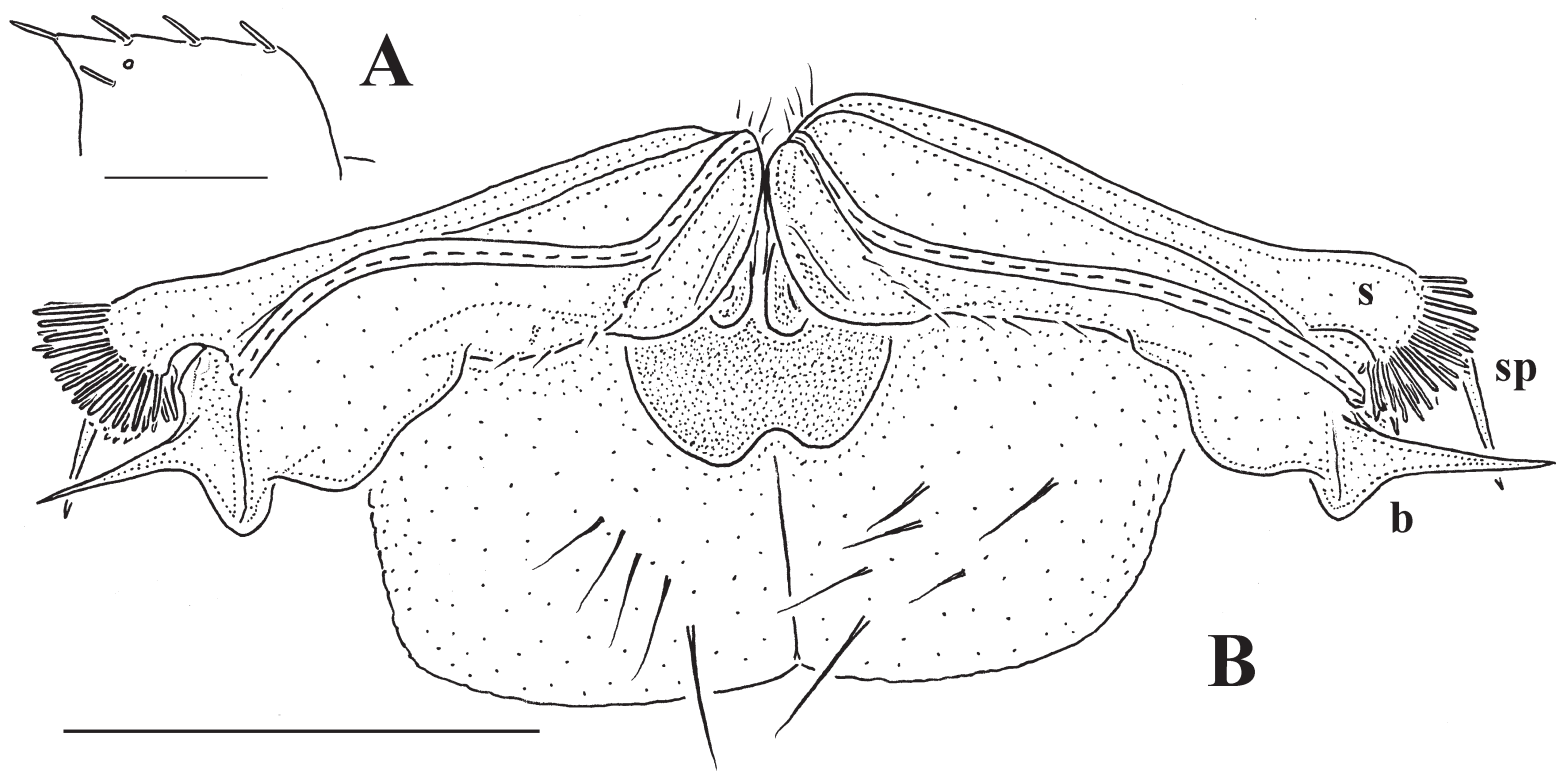
*Retrodesmus dammermani* Chamberlin, 1945, ♂ holotype from Java, Indonesia **A** left paratergite 10, dorsal view **B** both gonopods *in situ*, ventral view. – Scale bars: 0.2 mm.

**Figure 14.**
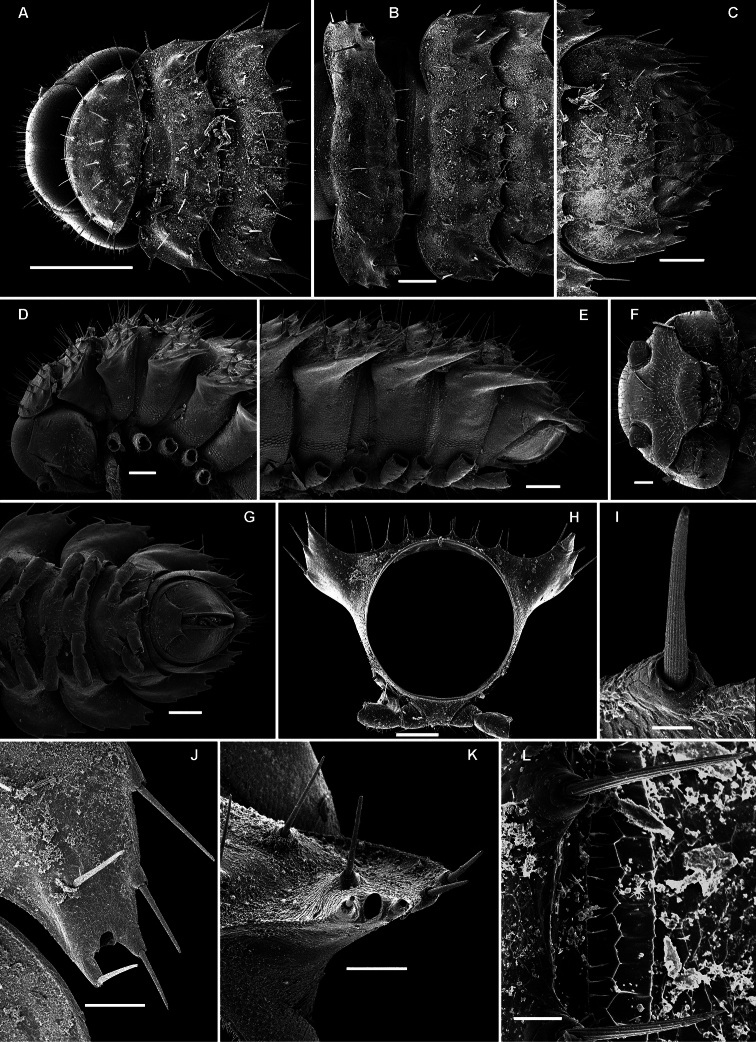
*Retrodesmus cavernicola* sp. n., ♀ subadult, paratype; **A, D** anterior body part, dorsal and lateral views, respectively **B** midbody segments, dorsal view **C, E, G** posterior body part, dorsal, lateral and ventral views, respectively **F** head, ventral view **H** cross-section of a midbody segment, caudal view **I** tergal seta **J, K** paratergite 15, dorsal and subcaudal views, respectively **L** limbus, dorsal view. – Scale bars: **A** 0.5 mm; **B–E, G, H** 0.2 mm; **F, J, K** 0.1 mm; **I, L** 0.02 mm.

**Figure 15.**
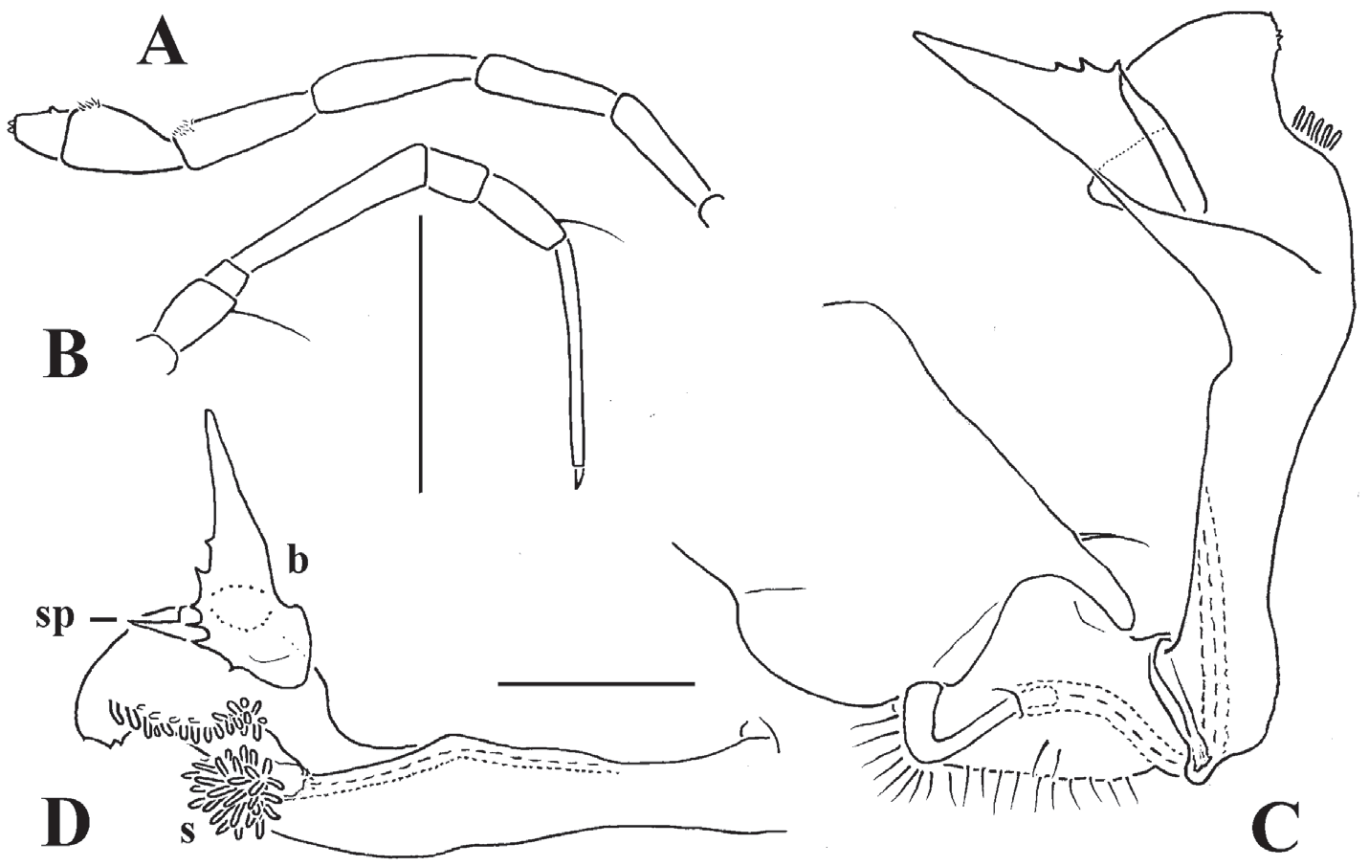
*Retrodesmus cavernicola* sp. n., ♂ paratype from Cave Bem Tem; **A** antenna, lateral view **B** midbody leg **C** left gonopod, submesal view **D** right gonopod, subfrontal view. – Scale bars: **A, B** 1.0 mm; **C, D** 0.2 mm.

**Figure 16.**
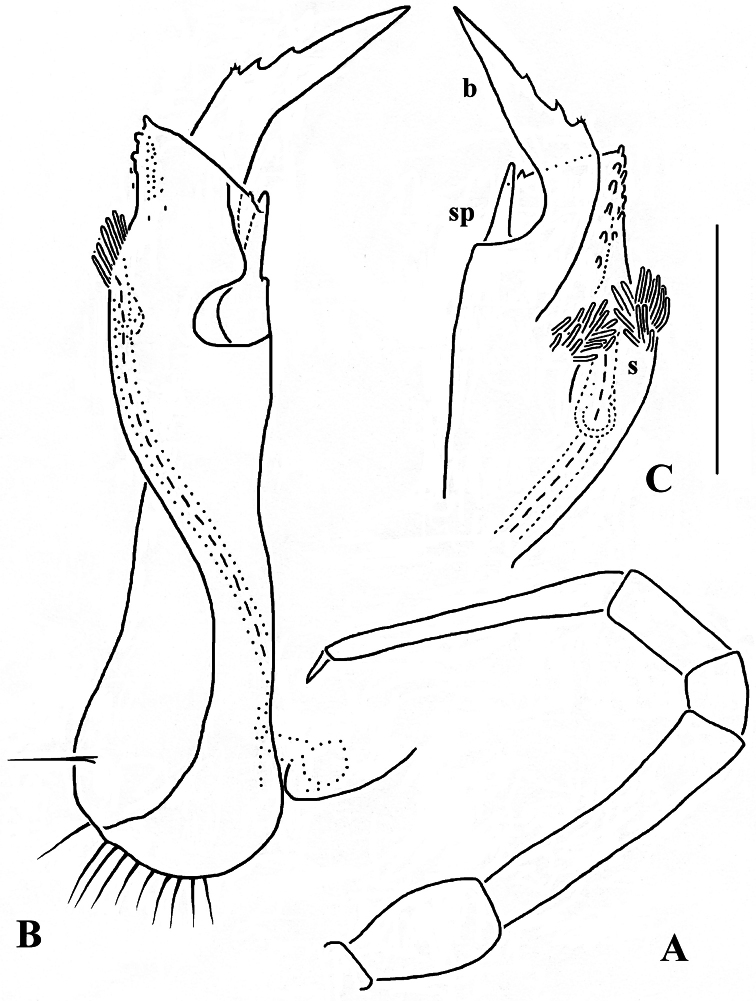
*Retrodesmus cavernicola* sp. n., ♂ holotype; **A** leg 9 **B, C** left gonopod, subdorsal and subventral views, respectively. – Scale bars: **A** 0.5 mm; **B, C** 0.2 mm.

The holotype of this species has been examined in order to shed light on the identity of both the genus and species. A new species is added as well. The differences between both are clear from Figs 12, 13 and Figs 14–16, as well as from the diagnosis of *Retrodesmus cavernicola* sp. n.

### Retrodesmus dammermani

Chamberlin, 1945

http://species-id.net/wiki/Retrodesmus_dammermani

Figs 12 , 13

Retrodesmus dammermani Holotype ♂ (AMNH), Java, Tjibodas, 1400 m, Aug. 1921, Dammerman [on label].

#### Descriptive notes and remarks.

The series also contains a microvial with several fragments of a presumed ♀ labeled “♀ allotype”, but, having not been mentioned in the original description (Chamberlin 1945), this ♀ cannot be considered as part of the type series.

The holotype, an intact ♂, has been restudied, with several colour pictures taken to show the habitus (Fig. 12), and line drawings executed of a midbody paratergite and the gonopods *in situ* (Fig. 13).

Chamberlin’s (1945) succinct description is basically correct in showing quite broad and mostly slightly upturned paraterga with 2 or 3 minute, lateral, setiferous incisions; the caudal corners of postcollum paraterga until the 17^th^ are produced increasingly well behind the rear tergal margin, roundly dentiform; the metaterga support three rather regular, transverse rows of short to medium-sized bacilliform setae; the ozopores are located rather close to the lateral margin of ozoporiferous paraterga, but quite far from the caudal corner (Fig. 13A). Body length ca 6 mm, width 0.55 mm.

The gonopods (Fig. 13B), contrary to Chamberlin’s (1945) sketch (his fig. 20), show only a slightly curved telopodite devoid of a drastic parabasal geniculation. The coxae bear several setae on the ventral side. The telopodite is rather stout, unipartite, slightly hollow on the caudal face, only subterminally subdivided into a frontal stump (**s**) beset with bacilliform ornamentations and surmounted by a long spine (**sp**), and a simple, similarly spinigerous branch (**b**). The seminal groove runs along the caudal face to flush open on the surface, with neither a solenomere nor an accessory seminal chamber, nor a hairy pulvillus, terminating near the base of both **s** and **b**.

As Hoffman (2005, p. 75) once put it quite sarcastically as regards the quality of Chamberlin’s (1945) paper, “There is no evidence that Professor Chamberlin invested much time in consultation of available literature sources”. Nevertheless, his *Retrodesmus* remains a valid genus sufficiently distinct from the other opisotretid genera.

In addition to the type species, *Retrodesmus* also includes *Retrodesmus cavernicola* sp. n., a presumed troglobite from Papua New Guinea.

### Genus Solaenaulus

Attems, 1940

http://species-id.net/wiki/Solaenaulus

#### Diagnosis.

A genus of Opisotretidae with 19 (♂) or 20 (♀) body segments. ♂ vertex without modifications. Metaterga with three regular, transverse rows of bacilliform setae. Frontolateral margin of midbody paraterga without shoulders. Ozopore usually lying very close to caudal margin of paratergite’s caudolateral corner (Figs 4C, D, F, 5B, G).

Gonopod telopodite elongate, subunciform, bipartite; basal process (**p**) on frontoventral face of femorite prominent, removed from femorite proper; distal part of telopodite usually beset with bacilliform ornamentations both over a prominent solenomere (**sl**) and an even more prominent exomere (**ex**); a small accessory seminal chamber present, but a hairy pulvillus absent (Figs 6A, C–F, 7).

#### Type species.

*Opisotretus butteli* Carl, 1922, by original designation of Attems (1940).

#### Remarks.

In addition to the type species, the genus currently contains only one known species: *Solaenaulus birmanicus* Carl, 1941 (Golovatch et al. 2010), which, however, is sometimes treated as a variety of the type species (Jeekel 2006).

### Descriptions of new species

#### Retrodesmus cavernicola sp. n.

urn:lsid:zoobank.org:act:F9034C5B-EC1B-4AD8-968D-7F77FF1AD0EB

http://species-id.net/wiki/Retrodesmus_cavernicola

Figs 14 –16

##### Type material.

Holotype ♂ (NMNHS), Papua New Guinea, Western Prov., Finim tel Plateau, Peep Hole Cave, 18.08.1975, leg. P. Beron (British Speleological Expedition).

Paratypes: 1 ♂, 1 ♀, 1 ♂ (incomplete), 5 juv. (17 segments) (NMNHS), 1 juv. (17 segments) (ZMUC), 1 ♀ subadult (19 segments) (MNHN JC 338), Papua New Guinea, Western Prov., Telefomin, Cave Bem Tem (No. 1), 31.07.1975, leg. British Speleological Expedition; 1 ♀ subadult (19 segments) (ZMUM), 1 ♀ subadult (19 segments) (SEM), 1 juv. (17 segments) (NMNHS), Finim tel Plateau, Upper Bitip Cave, west chamber, 21.11.1975, leg. British Speleological Expedition, FT-11; 1 ♀ (fragments) (NMNHS), Finim tel Plateau, bottom of a 150 m shaft near Girtoil, 08.08.1975, leg. British Speleological Expedition; 1 ♀ (fragments) (NMNHS), Chimbu Prov., Goglme Village, Cave Ogon I, 1975, leg. P. Beron (British Speleological Expedition).

##### Diagnosis.

Differs readily from *Retrodesmus dammermani* Chamberlin, 1945, the only other known species of *Retrodesmus*, by the particularly broad paraterga, several clearly troglomorphic features such as especially long and slender antennae, legs and metatergal setae, the latter also being very dense, the subcaudal position of the ozopores, and the shape and ornamentation of the gonopod apex.

##### Name.

To emphasize the obvious troglomorphic traits strongly suggesting obligate cave-dwelling; a noun in apposition.

##### Description.

Length of adults of both sexes ca 12 (♂) or 16 mm (♀), width of midbody pro- and metazona 0.95–1.0 and 2.0 mm (holotype and ♂ paratype) or 1.5 and 2.8 mm (♀ paratypes), respectively. Coloration in alcohol from uniformly pallid to light yellowish.

Body with 19 (♂) or 20 (♀) segments. Tegument mainly dull, at most slightly shining, texture very delicately alveolate. Head densely pilose throughout; epicranial suture superficial and thin; isthmus between antennae about twice the diameter of antennal socket. Antennae very long and slender, reaching behind segment 2 when stretched dorsally, geniculate between antennomeres 5 and 6, each latter with an apicodorsal group of tiny sensilla; antennomere 7 with a tiny mid-dorsal knob; antennomeres 2-6 subequal in length (Fig. 15A).

In width, collum << head < segment 2 < 3 = 4 < 5 (6) =15 (♂, ♀), thereafter body gradually tapering towards telson. Paraterga very strongly developed, starting from collum, invariably slightly to clearly upturned, set high, but always lying slightly below a faintly convex dorsum, with shoulders frontolaterally (Figs 14A–C). Caudal corner of postcollum paraterga invariably spiniform, pointed, extending increasingly behind rear tergal margin. Lateral edge of paraterga with 2 or 3 clear and deep setigerous indentations in poreless and poriferous segments, respectively. Pore formula normal; ozopores evident, round, flush open on dorsal surface, located very close to caudal margin at bottom of caudalmost lateral incision (Fig. 14J), lateral tooth being considerably shorter than medial one. Collum and each following metatergum with mostly 3+3 long, nearly pointed, but ribbed and subbacilliform setae arranged in three transverse, rather regular rows and borne on small stalks; polygonal bosses very flat; both rear rows of setae more irregular, placed very close to each other (Figs 14A–E, H). Stricture between pro- and metazona wide, shallow and smooth. Limbus very fine, microspiculate, the spikes mostly being rather sparse and irregular. Pleurosternal carinae absent (Figs 14D, E). Epiproct short, conical, directed caudoventrally; pre-apical papillae small (Figs 14C, E, G). Hypoproct trapeziform (Fig. 14G), setiferous papillae at caudal corners very small and well separated.

Sterna without modifications, rather broad, strongly setose (Fig. 14G). Epigynal ridge very low. Legs very long and slender, growing slightly slenderer towards telson (Figs 15B, 16A), ca 1.5 (♂) times as long as midbody height; femora and tarsi longest, subequal in length; sphaerotrichomes missing.

Gonopod aperture evident, transversely oblong-oval, taking up most of ventral part of metazonite 7. Gonopods (Figs 15C, D, 16B, C) with globose, medially fused coxae carrying a few setae on ventral face and a normal cannula mesally. Telopodite nearly straight, unipartite, rather short and stout. Distal part of telopodite split into a shorter frontal stump (**s**) (= solenomere?) beset with bacilliform ornamentations and a quite complex, subtriangular, pointed, caudal branch (**b**) with a spine (**sp**) at base. Seminal groove terminating near base of both **s** and **b**, with neither a distinct solenomere nor a hairy pulvillus, but with a small accessory seminal chamber.

##### Remarks.

Because of several apparent troglomorphic traits,this species seems to be a troglobite. Surprisingly, it appears to be rather widespread in western Papua New Guinea, occurring in places like Finim tel and Goglme which are separated from each other by >200 km.

It is the gonopod conformation, not the location of the ozopores, that clearly indicates the true affinities of *Retrodesmus cavernicola* sp. n. to *Retrodesmus dammermani*, despite the great geographical gap between Java and New Guinea that also separates these species.

#### Opisotretus beroni sp. n.

urn:lsid:zoobank.org:act:8B8C4BC4-80C2-4243-916A-057600056EFA

http://species-id.net/wiki/Opisotretus_beroni

Figs 17 –21

##### Type material.

Holotype ♂ (NMNHS), Papua New Guinea, Western Prov., Mount Fugilil, at camp, 2980 m a.s.l., 09.10.1975, leg. P. Beron (British Speleological Expedition).

##### Paratype.

1 ♀ subadult (19 segments) (ZMUC), same locality, together with holotype; 1 ♂, 1 ♀, 1 ♀ subadult (19 segments) (NMNHS), 1 ♂, 1 ♀ (ZMUM), 1 ♂ (SEM), same locality, Mount Fugilil, summit, 3150 m a.s.l., 29.09.1975, leg. P. Beron (British Speleological Expedition); 1 ♂, 1 ♀ (MNHN JC 339), Western Prov., Finim tel Plateau, Selminum doline, 2300 m a.s.l., forest litter, 02.10.1975, leg. Ph. Chapman & P. Beron (British Speleological Expedition); 1 ♀ (NMNHS), Papua New Guinea: Mount Wilhelm, Lake Pinde, 3480 m, 25.10.1975, leg. P. Beron (British Speleological Expedition); 1 ♂, 1 ♀ (NMNHS), 1 ♀ subadult (19 segments) (SEM), same locality, Mount Wilhelm, from 4260 m a.s.l. (14000 feet) to summit (4694 m a.s.l.), 24.10.1975, leg. P. Beron (British Speleological Expedition).

##### Diagnosis.

Differs readily fromcongeners by the shorter and bifid apical piece of the gonopod telopodite devoid of a spine level to a short solenomere, coupled with a deeper caudalmost incision of paraterga harbouring the ozopore in poriferous segments.

##### Name.

Honours Petar Beron (NMNHS), the principal collector of material.

##### Description.

Length of adults of both sexes ca 10-11 mm, width of midbody pro- and metazona 1.0 and 1.4 mm (holotype), 0.95 and 1.3 mm (♂ paratypes) or 1.1–1.2 and 1.5–1.6 mm (♀ paratypes), respectively. Coloration in alcohol from uniformly pallid to light yellowish.

Body with 19 (♂) or 20 (♀) segments. All characters like in *Retrodesmus cavernicola* sp. n., except as follows.

**Figure 17.**
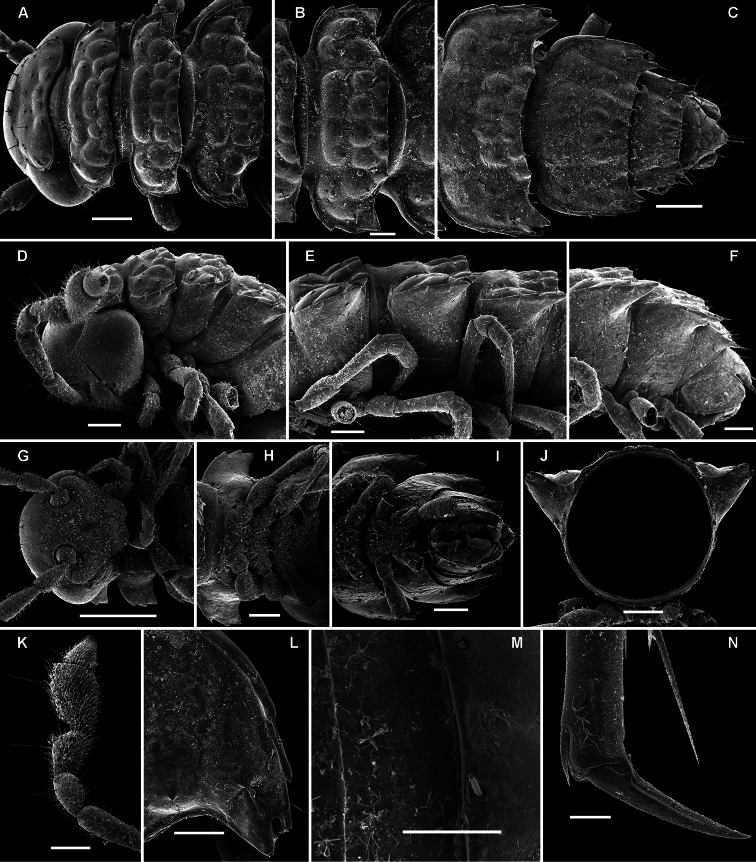
*Opisotretus beroni* sp. n., ♂ paratype from Mount Fugilil (summit); **A, D, G** anterior body part, dorsal, lateral and ventral views, respectively **B, E, H** midbody segments, dorsal, lateral and ventral views, respectively **C, F, I** posterior body part, dorsal, lateral and ventral views, respectively **J** cross-section of a midbody segment **K** antenna, lateral view **L** right paratergite 13, dorsal view **M** tergal setae **N** claw. – Scale bars: **G** 0.5 mm; **A, C–F, H–K** 0.2 mm; **B, L** 0.1 mm; **M** 0.05 mm; **N** 0.01 mm.

Antennae medium-sized, strongly clavate, extending behind segment 2 when stretched dorsally (Figs 17K, 20A).

In width, collum << segment 2 < 3 < head = 4 < 5 (6) =15 (♂, ♀), thereafter body gradually tapering towards telson. Paraterga of adults rather strongly developed, considerably smaller and set lower in ♀ subadults (Figs 17J, 19I), starting from collum, mostly subhorizontal to slightly declivous, set high, but always lying slightly below a faintly convex dorsum, with very faint shoulders frontolaterally (Figs 17A–C). Caudal corner of postcollum paraterga dentiform, narrowly rounded to nearly pointed, extending increasingly behind rear tergal margin only in a few caudalmost segments. Lateral edge of paraterga with 2 or 3 small setigerous indentations in poreless and poriferous segments, respectively. Ozopores evident, round, flush open on dorsal surface, located very close to caudal margin at bottom of caudalmost lateral incision (Figs 17C, L), lateral tooth being considerably shorter than medial one. Collum and each following metatergum with 3+3 very short bacilliform setae arranged in three regular transverse rows; polygonal bosses evident, transverse sulcus superficial (Figs 17A–F).

Sterna without modifications, rather broad, strongly setose (Fig. 17H). Epigynal ridge very low. Legs rather long, clearly incrassate in ♂ (Figs 17D–F), ca 1.2–1.3 (♂) or 1.0–1.1 times (♀, juveniles) as long as midbody height; femora and tarsi longest, subequal in length; sphaerotrichomes missing, but ♂ prefemora and femora beset with short spiniform setae ventrally (Fig. 20B).

**Figure 18.**
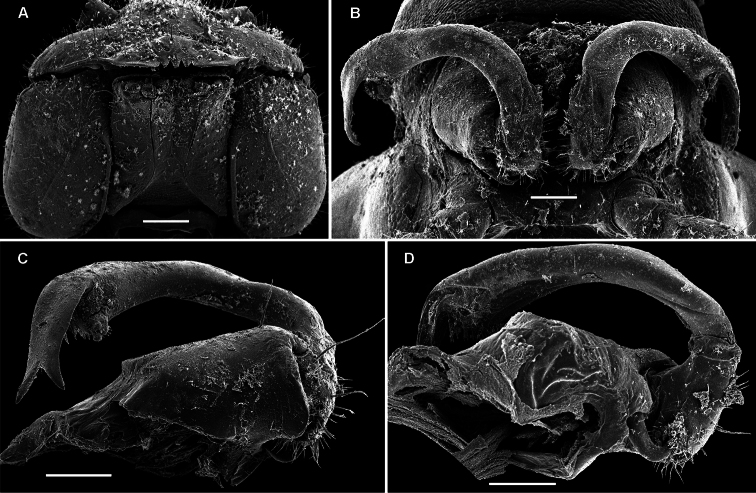
*Opisotretus beroni* sp. n., ♂ paratype from Mount Fugilil (summit); **A** head, ventral view **B** both gonopods *in situ*, ventral view **C, D** left gonopod, sublateral and submesal views, respectively. – Scale bars: 0.01 mm.

Gonopod telopodite (Figs 18B–D, 20C, D, 21) only slightly curved, unipartite, rather long and slender; apical piece (**a**) distal to a very short solenomere (**sl**) elongate, more strongly curved, clearly bifid, on caudal face with a few to several denti- or spiniform ornamentations, but devoid of a strong parabasal spine level to **sl**. An accessory seminal chamber at base of **sl** evident, crowned with a hairy pulvillus.

**Figure 19.**
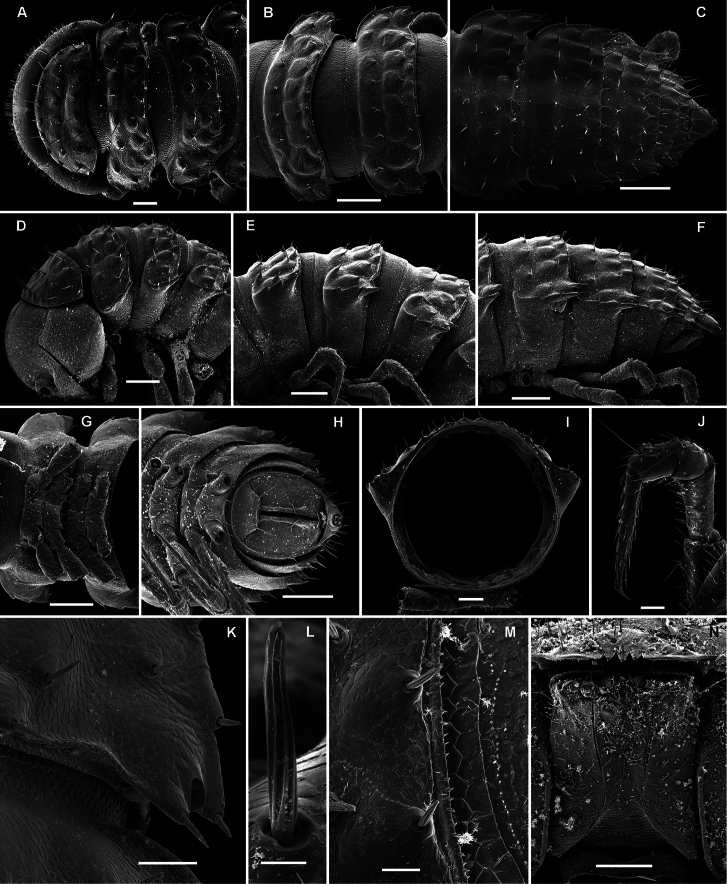
*Opisotretus beroni* sp. n., ♀ subadult, paratype from Mount Fugilil (summit); **A, D** anterior body part, dorsal and lateral views, respectively **B, E, G** midbody segments, dorsal, lateral and ventral views, respectively **C, F, H** posterior body part, dorsal, lateral and ventral views, respectively **I** cross-section of a midbody segment **J** midbody leg **K** right paratergite 13, dorsal view **L, M** tergal setae **N** gnathochilarium, ventral view. – Scale bars: **B–H**,0.2 mm; **A, I, N** 0.1 mm; **J, K** 0.05 mm; **M** 0.02 mm; **L** 0.01 mm.

**Figure 20.**
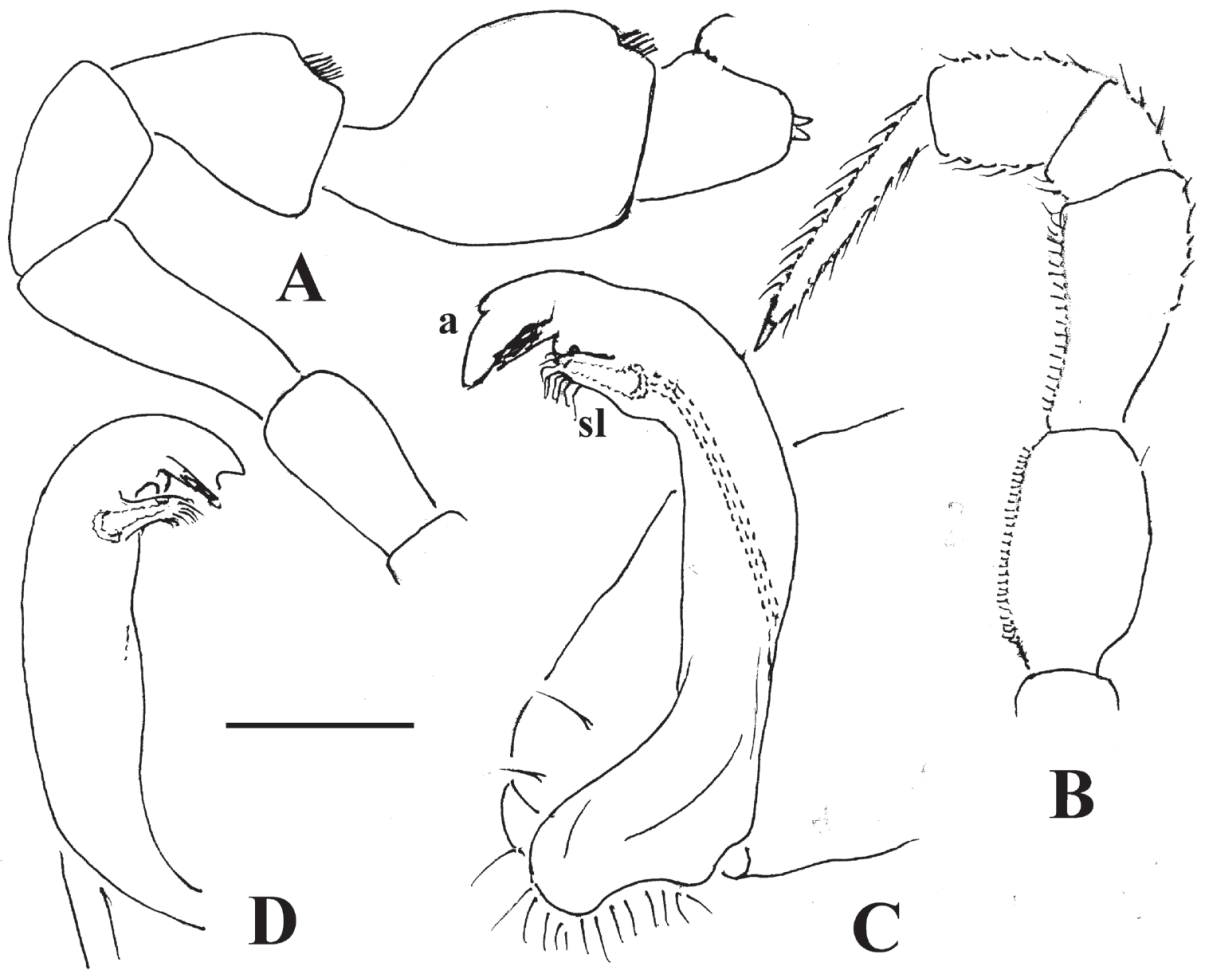
*Opisotretus beroni* sp. n., ♂ paratype from Mount Fugilil (summit); **A** antenna, lateral view **B** midbody leg **C, D** left gonopod, sublateral and submesal views, respectively. – Scale bar: 0.2 mm.

##### Remarks.

**Figure 21.**
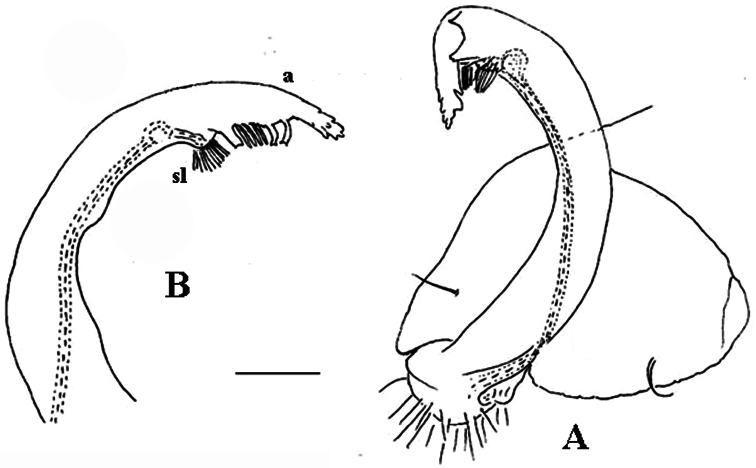
*Opisotretus beroni* sp. n., ♂ paratype from Selminum doline; **A, B** left gonopod, submesal and sublateral views, respectively. – Scale bar: 0.2 mm. After Golovatch et al. (2010).

The gonopod structure of this new species has already been illustrated by mistake elsewhere (Golovatch et al. 2010, figs 78 & 80), in connection with documenting the record of *Solaenaulus butteli* in Papua New Guinea. The same drawings (Fig. 21) are reproduced here again.

#### Opisotretus hagen sp. n.

urn:lsid:zoobank.org:act:9CD9B717-7849-4122-B437-ABCF2AC79C48

http://species-id.net/wiki/Opisotretus_hagen

Figs 22 , 23

##### Type material.

Holotype ♂ (NMNHS), Papua New Guinea, Western Highlands Prov., Mount Hagen, ca 1990 m a.s.l., in town, 22.10.1975, leg. P. Beron (British Speleological Expedition).

##### Paratypes.

1 ♀ subadult (19 segments), 1 juv. (fragments) (NMNHS), 1 ♀ subadult (19 segments) (SEM; MNHN JC 340), same locality, together with holotype.

##### Diagnosis.

Differs readily fromcongeners by a modified ♂ head (two prominent paramedian tubercles above the antennal sockets), coupled with a less strongly curved, nearly suberect gonopod telopodite, with its apical piece crowned with several peculiar, mostly digitiform outgrowths.

##### Name.

Referring to the type locality; a noun in apposition.

##### Description.

Length of holotype (and of subadult ♀ paratypes) ca 9 mm, width of midbody pro- and metazona 0.8 and 1.15 mm, respectively. Coloration in alcohol from uniformly pallid to light yellowish.

Body with 19 (♂) or 20 (♀) segments. All characters like in *Retrodesmus cavernicola* sp. n., except as follows.

**Figure 22.**
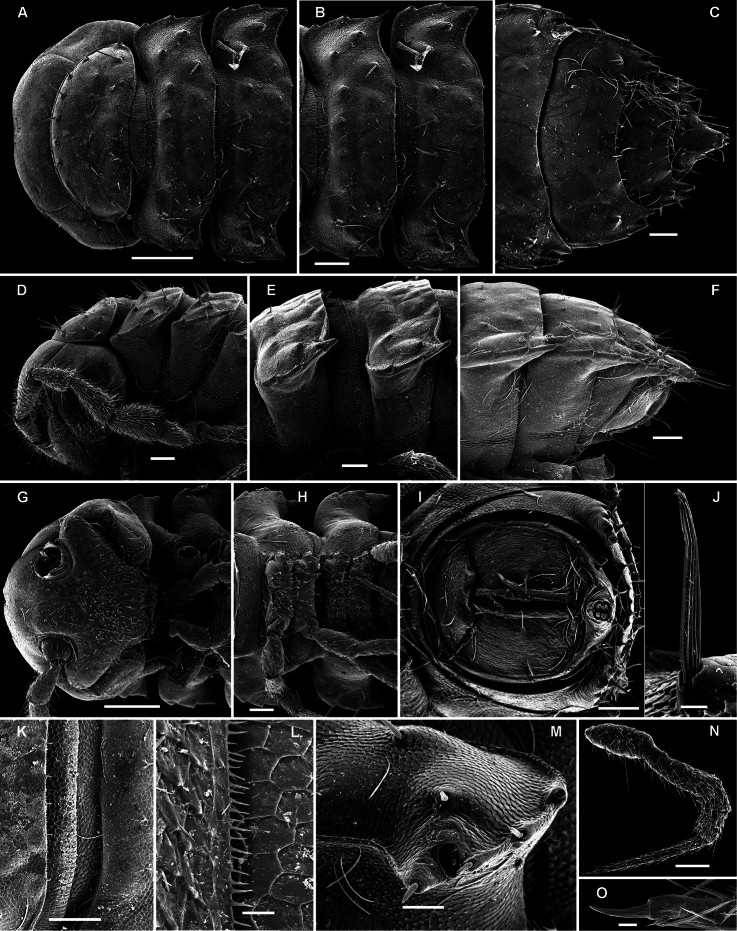
*Opisotretus hagen* sp. n., ♀ subadult, paratype; **A, D, G,** anterior body part, dorsal, lateral and ventral views, respectively **B, E, H** midbody segments, dorsal, lateral and ventral views, respectively **C, F, I** posterior body part, dorsal, lateral and caudal views, respectively **J** tergal seta, lateral view **K** tegument texture, dorsal view **L** limbus, dorsal view **M** midbody paratergite, subcaudal view **N** midbody leg; **O,** claw. – Scale bars: **A, G** 0.2 mm; **B–F, H, I, K, N** 0.1 mm; **M** 0.05 mm; **J, L, O** 0.01 mm.

**Figure 23.**
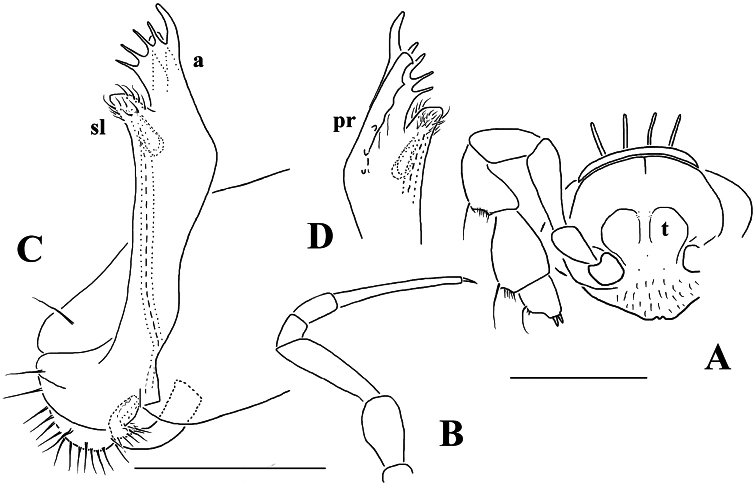
*Opisotretus hagen* sp. n., ♂ holotype; **A** head, frontal view **B** midbody leg **C, D** right gonopod, mesal and lateral views, respectively. – Scale bars: **A** 1.0; **B** 0.5 mm; **C, D** 0.2 mm.

♂ head with two round, paramedian, rather high tubercles (**t**) above antennal sockets (Fig. 23A). Antennae medium-sized, strongly clavate, extending behind segment 2 when stretched dorsally (Figs 22D, 23A).

In width, collum << segment 2 < head = 3 < 4 < 5 (6) =15 (♂), thereafter body gradually tapering towards telson. Paraterga rather strongly developed, starting from collum, mostly subhorizontal to slightly declivous, set high, but always lying slightly below a moderately (♀, juv.) to weakly (♂) convex dorsum, with rather faint shoulders frontolaterally (Figs 22A–C). Caudal corner of postcollum paraterga dentiform, always pointed and extending increasingly well behind rear tergal margin. Lateral edge of paraterga with 2 or 3 small setigerous indentations in poreless and poriferous segments, respectively. Ozopores evident, round, flush open on dorsal surface, located very close to caudal margin at bottom of caudalmost lateral incision (Figs 22C, E, M), lateral tooth being very considerably shorter than medial one. Collum and each following metatergum with 3+3 long bacilliform setae arranged in three regular transverse rows; polygonal bosses flat, but visible (Figs 22A–F).

Sterna without modifications, rather broad, strongly setose (Fig. 22H). Legs long, incrassate in ♂ due to prefemora alone (Figs 22N, 23B), ca 1.5 times (♂) as long as midbody height; femora and tarsi longest, subequal in length; sphaerotrichomes or other modified setae missing.

Gonopod telopodite (Figs 23C, D) only very slightly curved, nearly suberect, unipartite, rather long and slender; apical piece (**a**) distal to a very short solenomere (**sl**) rather short, on caudal face with a few finger-shaped ornamentations and a strong, parabasal, subspiniform process (**pr**). An accessory seminal chamber at base of **sl** evident, crowned with a hairy pulvillus.

##### Remarks.

This is the first *Opisotretus* showing ♂ head modifications, thus confirming the character as being only species-specific (Golovatch 1988).

#### Opisotretus deharvengi sp. n.

urn:lsid:zoobank.org:act:3BE21451-287B-47B4-BE42-0CBB5FC8AB8D

http://species-id.net/wiki/Opisotretus_deharvengi

Figs 24 , 25

##### Type material.

Holotype ♂ (MZB), Indonesia, Sulawesi Selatan, Bone (Watampone), Taccipi, Cave GuaKarabice, inside cave, hand collection, 30.07.1989, leg. L. Deharveng (SULS-068).

##### Paratype.

1 ♀ (SEM; MNHN JC 341), same locality, together with holotype.

##### Diagnosis.

Differs readily fromcongeners both by tergal sculpture and lateral paratergal incisions being rather poorly developed, coupled with the presence of a short solenomere and a peculiar ornamentation in the apical piece (**a**) of the gonopod telopodite.

##### Name.

Honours Louis Deharveng, the collector.

##### Description.

Length of holotype ca 9 mm, width of midbody pro- and metazona 0.7 and 1.0 mm, respectively. Coloration in alcohol uniformly pallid.

Body with 19 (♂) or 20 (♀) segments. All characters like in *Retrodesmus cavernicola* sp. n., except as follows.

Antennae broken off, but likely long and slender.

**Figure 24.**
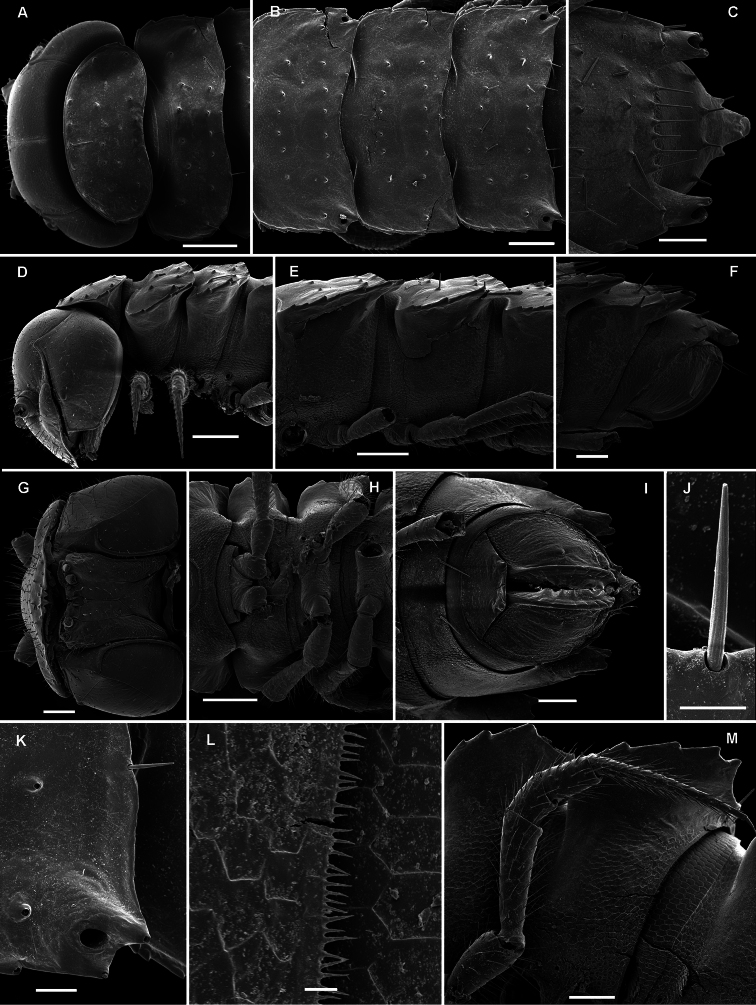
*Opisotretus deharvengi* sp. n., ♀ paratype; **A, D** anterior body part, dorsal and lateral views, respectively **B, E, H** midbody segments, dorsal, lateral and ventral views, respectively **C, F, I** posterior body part, dorsal, lateral and caudal views, respectively **G** head ventral view **J** tergal seta, lateral view **K** left paratergite 13, dorsal view **L** tegument texture and limbus, dorsal view **M** midbody paratergite and leg *in situ*, ventrolateral view. – Scale bars: **A, B, D, E, H** 0.2 mm; **C, F, G, I, M** 0.1 mm; **K** 0.05 mm; **L** 0.01 mm.

In width, collum << segments 2-4 < 5 < 6=15 < head (♂); after 15^th^, body gradually tapering towards telson. Paraterga strongly developed, starting from a kidney-shaped collum, mostly subhorizontal, largely set high, almost level to (♂) or only very slightly below a weakly convex dorsum (♀), with faint shoulders frontolaterally (Figs 24A–C). Caudal corner of postcollum paraterga dentiform, always narrowly rounded and extending increasingly well behind rear tergal margin only in segments 16-18 (19). Lateral edge of paraterga with 2 or 3 small setigerous indentations in poreless and pori-ferous segments, respectively. Ozopores evident, round, flush open on dorsal surface, located very close to caudal margin at bottom of caudalmost lateral incision (Fig. 24B, C, I, K), lateral tooth being only slightly shorter than medial one. Collum and each following metatergum with 3+3 long bacilliform setae arranged in three regular transverse rows; polygonal bosses flat, but visible (Figs 24A–F).

Legs long and very slender (Fig. 24M), ca 2.0-2.1 (♂) or 1.5-1.6 times (♀) as long as midbody height; ♂ prefemora not incrassate, femora and tarsi longest, subequal in length, but tarsi especially slender; sphaerotrichomes or other modified setae missing.

**Figure 25.**
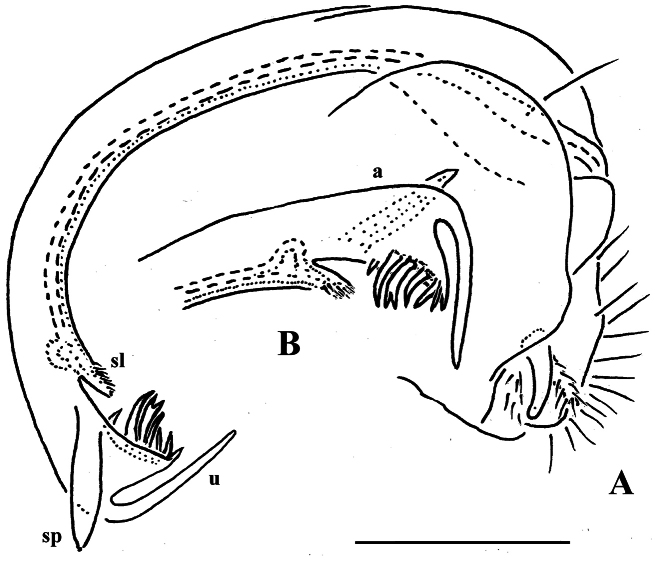
*Opisotretus deharvengi* sp. n., ♂ holotype; **A, B** left gonopod, subventral and subdorsal views, respectively. – Scale bar: 0.1 mm.

Gonopod telopodite (Fig. 25) clearly curved, unipartite, long and slender; apical piece (**a**) distal to a short solenomere (**sl**) rather long due to a terminal uncus (**u**) bearing near its base a strong subcaudal spine (**sp**) and a short field of subspiniform, mostly curved ornamentations. An accessory seminal chamber at base of **sl** evident, crowned with a hairy pulvillus.

##### Remarks.

This is the first formal encounter of an *Opisotretus* species in Sulawesi, Indonesia. Due to its long legs and uncoloured tegument, *Opisotretus deharvengi* sp. n. is likely to represent a troglobite. *Opisthoporodesmus bacillifer*, the only other opisotretid known from Sulawesi, differs readily in having only two, not three, lateral incisions on the paraterga (Carl 1912).

#### Opisotretus spinosus sp. n.

urn:lsid:zoobank.org:act:155B2375-F1DF-46D8-A194-3FD75766B75D

http://species-id.net/wiki/Opisotretus_spinosus

Fig. 26

##### Type material.

Holotype ♂ (MZB), Indonesia, Java, Jawa Tengah, Cilicap, Nusakambangan Island, near Cave Goa Kali Empat, litter, sieving and Berlese extraction, 19.02.2011, leg. L. Deharveng & Dito (JAVA-NK32).

##### Paratype.

1 ♀ (MNHN JC 342), same locality, together with holotype.

##### Diagnosis.

Differs readily fromcongeners by the presence of a clear, bare hump on the ♂ vertex, coupled with the presence both of only a rudimentary solenomere and a peculiar spination of the apical piece (**a**) of the gonopod telopodite.

##### Name.

To emphasize the highly spinose apical piece of the gonopod telopodite.

##### Description.

Length of holotype ca 4 mm, width of midbody pro- and metazona 0.3 and 0.5 mm, respectively. Length of paratype ca 5 mm, width of midbody pro- and metazona 0.45 and 0.6 mm, respectively. Coloration in alcohol uniformly pallid.

Body with 19 (♂) or 20 (♀) segments. All characters like in *Retrodesmus cavernicola* sp. n., except as follows.

**Figure 26.**
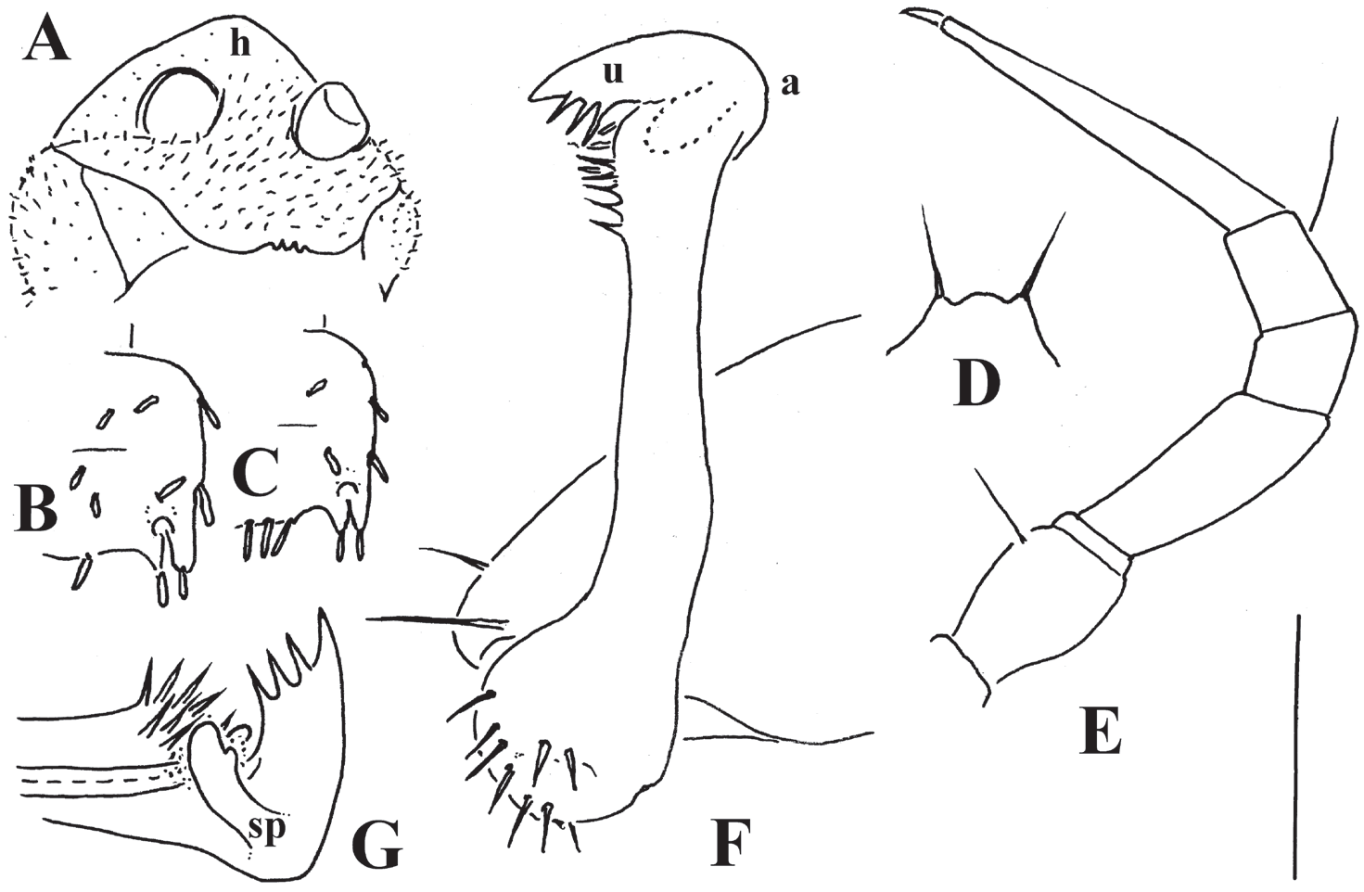
*Opisotretus spinosus* sp. n., ♂ holotype; **A** head, frontolateral view **B, C** right paratergites 15 and 18, respectively, dorsal view **D** hypoproct, ventral view **E** midbody leg **F, G** right gonopod, subventral and subdorsal views, respectively. – Scale bar: **A–C** 0.2 mm; **D, E** 0.1 mm; **F, G** 0.05 mm.

♂ head with an evident, bare, rounded vertigial hump (Fig. 26A, **h**). Antennae broken off, but obviously medium-sized.

In width, collum << segments 2 & 3 < head = 4 ≤ 5 < 6=15 < (♂, ♀); thereafter body gradually tapering towards telson. Paraterga rather poorly developed (Figs 26B, C), starting from a subcordiform, broadly rounded collum, mostly faintly declivous and continuing the outline of a quite convex dorsum (especially so in ♀), largely set rather high, at about ¼ to 1/3 of midbody height, with faint shoulders frontolaterally (Figs 26B, C). Caudal corner of postcollum paraterga dentiform, always narrowly rounded and extending increasingly well behind rear tergal margin in segments 12-18 (♂) or 15-19 (♀). Lateral edge of paraterga with 2-3 or 3-4 small setigerous indentations in poreless and poriferous segments, respectively. Ozopores evident, round, flush open on dorsal surface, lying clearly in front of caudal margin at bottom of caudalmost lateral incision, both lateral and medial teeth being subequal (Figs 26B, C). Collum and each following metatergum with 3+3, rather long, bacilliform setae arranged in three regular transverse rows; polygonal bosses flat, poorly visible. Hypoproct subtrapeziform, as in Fig. 26D.

Legs rather short and stout, ca 1.2-1.3 (♂) or 1.0-1.1 times (♀) as long as midbody height; tarsi longest and particularly slender (Fig. 26E), sphaerotrichomes or other modified setae missing.

Gonopod telopodite (Figs 26F, G) clearly curved, unipartite, long and slender; apical piece (**a**) distal to a vestigial solenomere strongly curved due to a terminal uncus (**u**) bearing near its base a strong subcaudal spine (**sp**) and a field of spiniform ornamentations. An accessory seminal chamber at base of solenomere rather evident, but probably devoid of a hairy pulvillus.

##### Remarks.

This new *Opisotretus* species has been taken together with several immature females (18 segments) of a different, somewhat larger and slightly pigmented (reddish metaterga and antennae) opisotretid with somewhat broader paraterga and a different location of the ozopores (these being placed close to the caudal tergal margin) which could not be identified in the absence of adult male material.

#### Martensodesmus cattienensis sp. n.

urn:lsid:zoobank.org:act:6B037C95-B3CB-4016-80C3-68CFB46E1BF2

http://species-id.net/wiki/Martensodesmus_cattienensis

Figs 27 –29

Martensodesmus sp. – Golovatch et al. 2011b: 81.

##### Type material.

Holotype ♂ (MNHN JC 343), Vietnam, Dongnai Prov., Cat Tien National Park, lowland semi-deciduous tropical monsoon forest, ca 150 m a.s.l., 107°10'–107°34'E, 11°21'–11°48'N, 08-22.11.2005, leg. A. E. Anichkin.

##### Paratypes.

1 ♂, 1 ♀ (MNHN JC 343), 1 ♂, 1 ♀, 1 ♀ fragment (ZMUM), 1 ♂ (ZMUC), same locality, together with holotype; 1 ♂, 1 ♀, 1 ♀ subadult (MNHN JC 343), 1 ♂, 1 ♀ (SEM), 1 ♂, 1 ♀, 1 ♀ subadult (ZMUM), same locality, 01.06.2005, leg. A. E. Anichkin; 1 ♂ (MNHN JC 343), 1 ♂ (NMNHS) same locality, 15.07.2005, leg. A. E. Anichkin.

##### Diagnosis.

Differs readily fromcongeners by the missing modifications on the ♂ vertex, coupled with quite well developed shoulders on metaterga, the high and broad paraterga, as well as the presence near the gonopod telopodite’s midpoint of three strong spines proximally to a considerably attenuating acropodite.

##### Name.

To emphasize the type locality.

##### Description.

Length of holotype ca 5.5 mm, width of midbody pro- and metazona ca 0.8 and 1.0 mm, respectively. Length of ♂ paratypes ca 5.5–6.0 mm, width of midbody pro- and metazona ca 0.75–0.8 and 1.0–1.1 mm, respectively. Length of ♀ paratypes ca 7.0-8.0 mm, width of midbody pro- and metazona ca 0.9–0.95 and 1.1–1.15 mm, respectively. Coloration in alcohol from nearly uniformly light yellowish to head and several anterior segments slightly infuscate, light yellow-brown, more rarely with a rusty reddish tint.

Body with 19 (♂) or 20 (♀) segments. All characters like in *Retrodesmus cavernicola* sp. n., except as follows.

**Figure 27.**
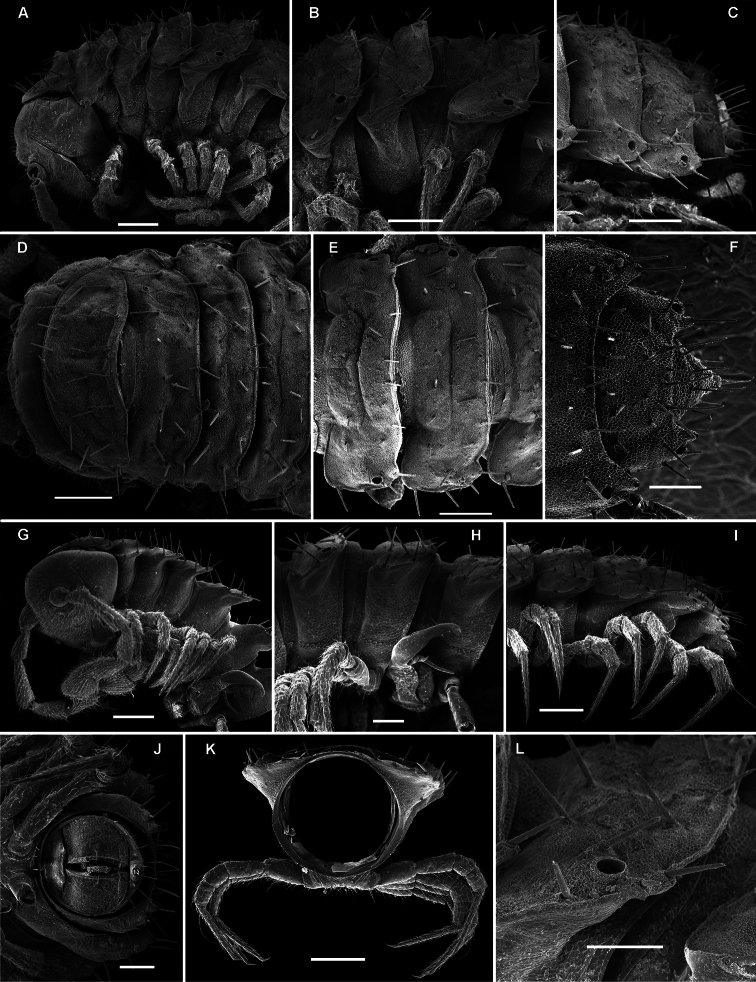
*Martensodesmus cattienensis* sp. n., ♂ paratype; **A, D, G** anterior body part, lateral, dorsal and ventrolateral views, respectively **B, E** midbody segments, lateral and dorsal views, respectively **C, F, I, J** posterior body part, dorsolateral, dorsal, lateral and caudal views, respectively **H** segments 5-7 with an exposed left gonopod, lateral view **K** cross-section of a midbody segment, caudal view **L** midbody paratergite with setae and an ozopore, lateral view. – Scale bars: **A–E, G, I, K** 0.2 mm; **F, H, J, L** 0.1 mm.

Antennae very long, but strongly clavate (Fig. 27G), extending behind segment 4 (♂) or 3 (♀) when stretched dorsally.

In width, collum << head = segments 2 & 3 < 4 < 5 < 6=15 (16); thereafter body gradually tapering towards telson. Paraterga well-developed (Fig. 27), starting from a broadly rounded, kidney-shaped collum, mostly only very faintly declivous to continue the outline of a rather slightly convex dorsum, largely set high, at about ¼ of midbody height, with quite strong shoulders frontolaterally (Figs 27D, E). Caudal corner of postcollum paraterga mostly dentiform, always narrowly rounded and extending increasingly well behind rear tergal margin only in segments 15-18 (♂) or 16-19 (♀). Lateral edge of paraterga with 2 or 3 small setigerous indentations in poreless and poriferous segments, respectively. Ozopores very evident, round, flush open on dorsal surface, clearly removed from caudal margin and lying anteriorly to bottom of caudalmost lateral incision (Figs 27A–C, E, F, H, I, L), lateral tooth being clearly shorter than medial one. Each postcollum metatergum with 3+3, long, bacilliform setae arranged in three regular transverse rows; polygonal bosses flat, but visible, while transverse sulcus mostly rather deep (Figs 27A–I).

**Figure 28.**
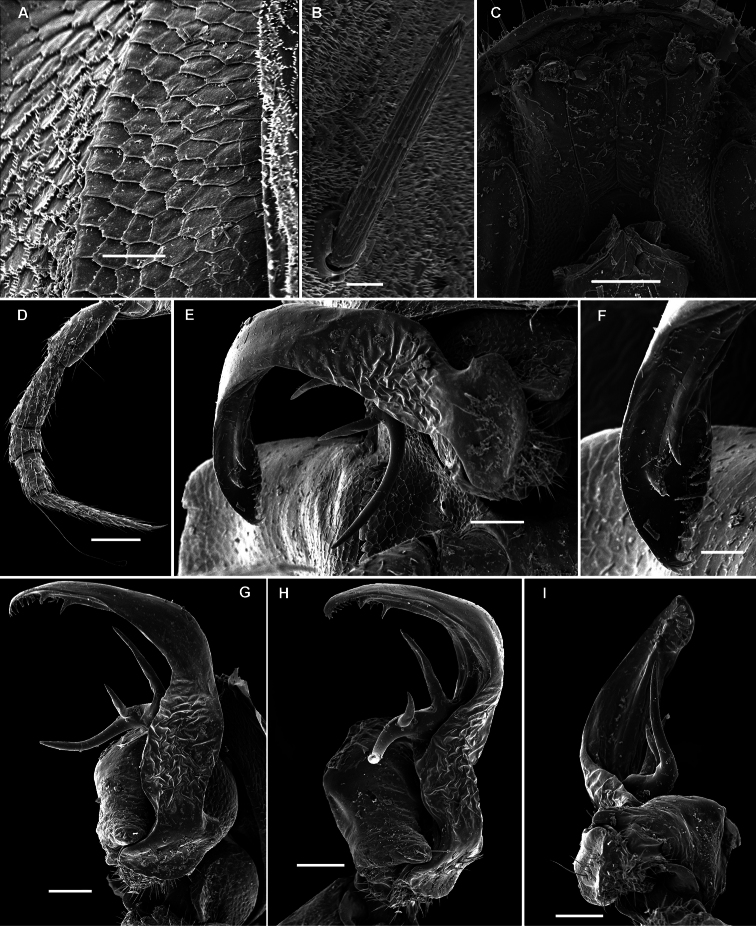
*Martensodesmus cattienensis* sp. n., ♀ (**A–F**) & ♂ (**G–L**) paratypes; **A** tegument texture, dorsal view **B** tergal seta, sublateral view **C** gnathochilarium, ventral view **D** midbody leg **E** right gonopod *in situ*, ventral view **F** tip of gonopod telopodite, ventral view **G–I** dissected left gonopod, subdorsal, frontal and submesal views, respectively. – Scale bars: **C, D** 0.1 mm; **E, G–I** 0.05 mm; **A, F** 0.02 mm; **B** 0.01 mm.

Legs rather long and slender, ca 1.4–1.5 (♂) or 1.2–1.3 (♀) times as long as midbody height (♂); tarsi longest and particularly slender (Figs 27A, B, G–I, K, 28D), sphaerotrichomes or other modified setae missing.

**Figure 29.**
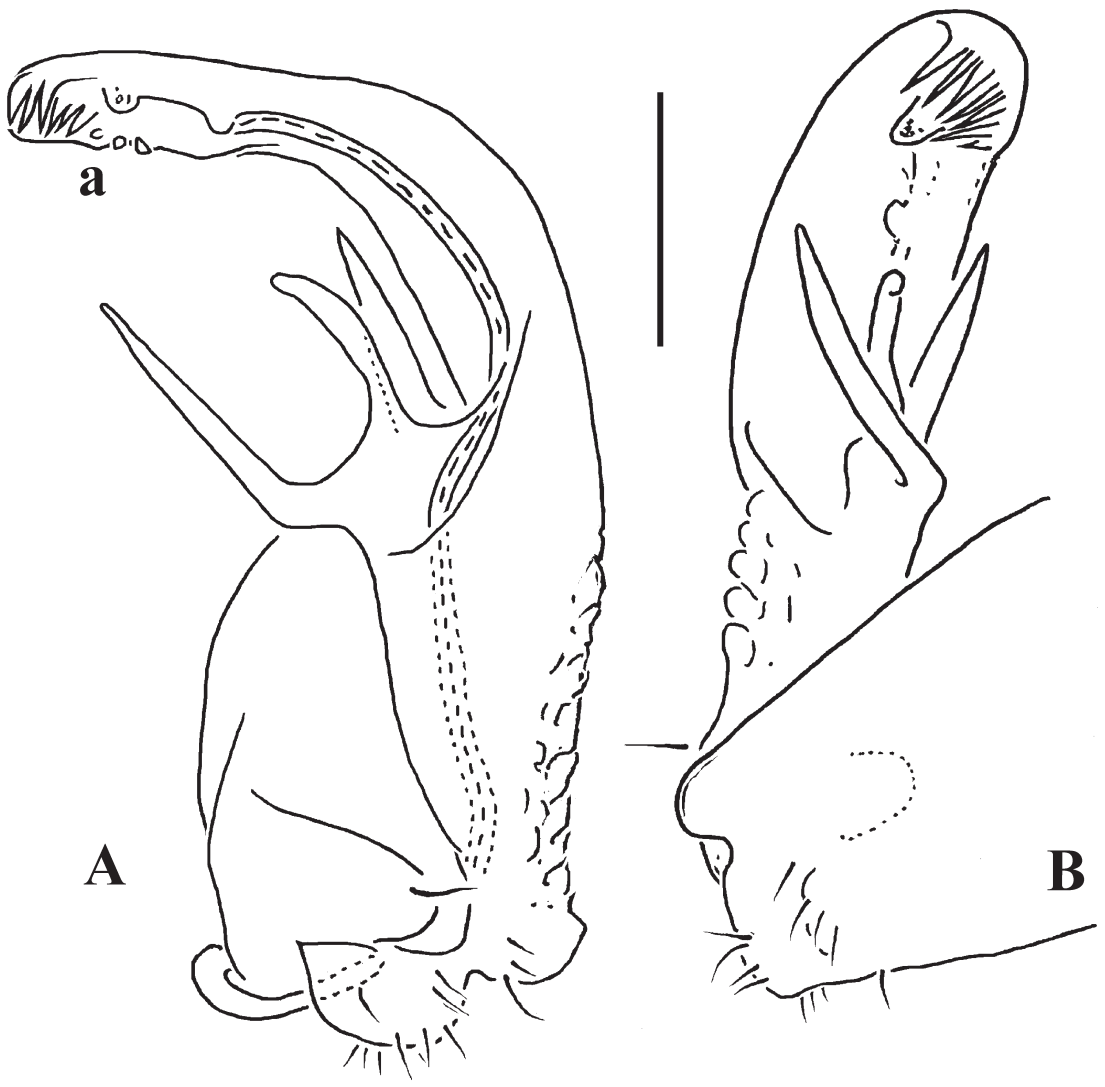
*Martensodesmus cattienensis* sp. n., ♂ paratype; **A, B** right gonopod, subventral and subfrontal views, respectively. – Scale bar: 0.1 mm.

Gonopod telopodite (Figs 28E–I, 29) clearly curved, but stout, unipartite; basal half voluminous and supplied with three strong spines, distal half gradually attenuating, apical piece (**a**) distal to a vestigial solenomere with a number of short spinules. Neither bacilliform ornamentations nor an accessory seminal chamber, nor a hairy pulvillus.

##### Remarks.

This species has already been referred to as *Martensodesmus* sp. elsewhere (Golovatch et al. 2011b).

#### Martensodesmus bedosae sp. n.

urn:lsid:zoobank.org:act:4CE2CCEE-F30A-4E9D-88FE-757C874FD3E8

http://species-id.net/wiki/Martensodesmus_bedosae

Figs 30 –32

##### Type material.

Holotype ♂ (IZAS), China, Guangxi, Hechi County, Duan Xian, Baling karst hill, disturbed forest, 109.07333°E, 23.98171°N, litter, sieving and Berlese extraction, 26.04.2010, leg. L. Deharveng & A. Bedos (CHIgx10-61).

##### Paratypes.

1 ♂ fragmented, 1 ♀ fragment (SCAU), 1 ♂, 1 ♀ (MNHN JC 344), 1 ♂ (SEM), same locality, together with holotype.

##### Diagnosis.

Differs readily fromcongeners by the presence of a well-developed frontobasal process **p** on the ventral side of the gonopod femorite, coupled with no modifications on the ♂ vertex.

##### Name.

Honours Anne Bedos, one of the collectors.

##### Description.

Length of holotype and ♂ paratypes ca 4.0 mm, width of midbody pro- and metazona ca 0.4 and 0.6 mm, respectively. Length of ♀ paratypes ca 5.5 mm, width of midbody pro- and metazona ca 0.6-0.65 and 0.8-0.9 mm, respectively. Coloration in alcohol from nearly uniformly pallid to head, collum and following metazona (especially their caudal halves) light rusty brown, anterior body portion in ♂♂ being clearly more infuscate, rusty brown, compared to ♀♀.

Body with 19 (♂) or 20 (♀) segments. All characters like in *Retrodesmus cavernicola* sp. n., except as follows.

Antennae medium-sized, strongly clavate, extending behind segment 3 (♂, broken off in the sole complete ♀) when stretched dorsally.

**Figure 30.**
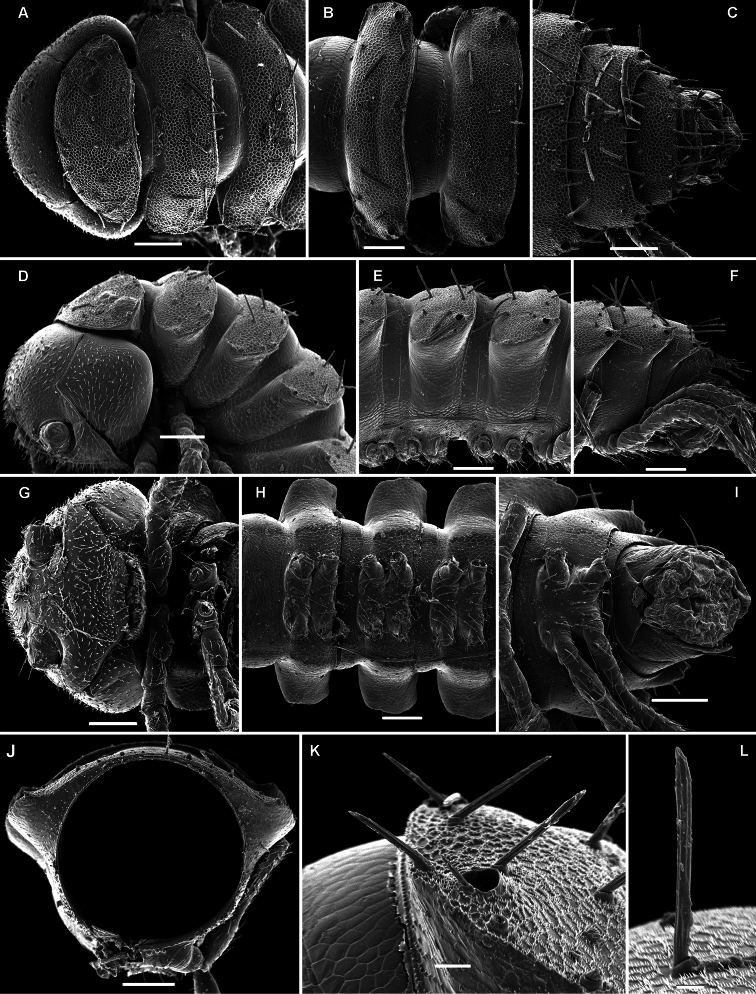
*Martensodesmus bedosae* sp. n., ♂ paratype; **A, D, G** anterior body part, dorsal, lateral and ventral views, respectively **B, E, H** midbody segments, dorsal, lateral and ventral views, respectively **C, F, I** posterior body part, dorsal, lateral and ventral views, respectively **J** cross-section of a midbody segment, caudal view **K** midbody paratergite with setae and an ozopore, lateral view **L** tergal seta, lateral view. – Scale bars: **A–J** 0.1 mm; **K** 0.02 mm; **L** 0.01 mm.

**Figure 31.**
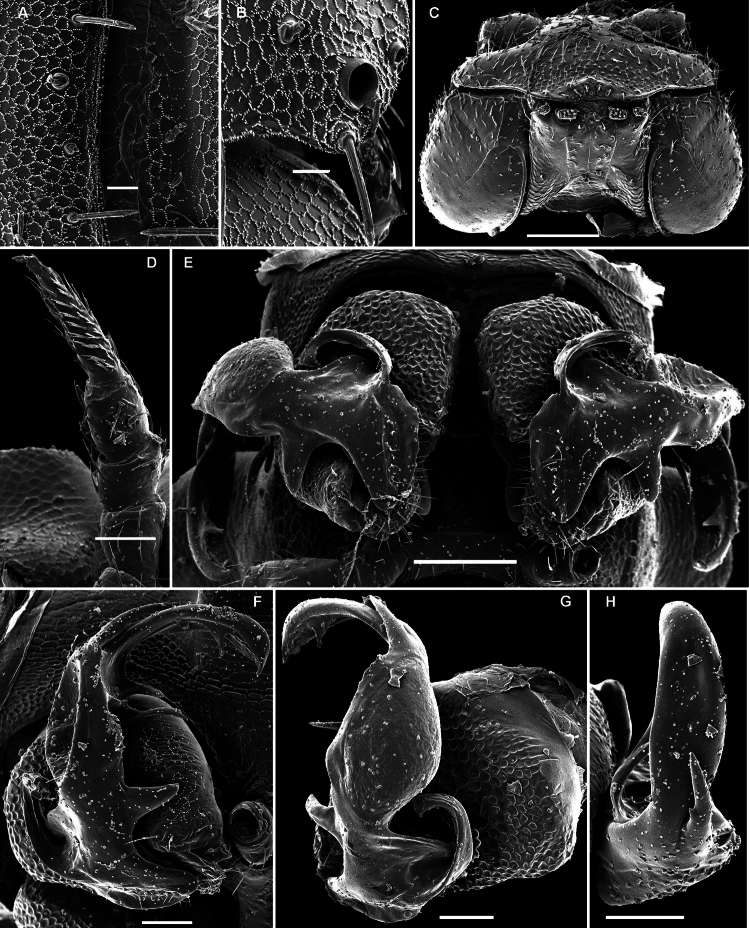
*Martensodesmus bedosae* sp. n., ♂ paratype; **A, B** tegument texture, tergal setae and ozopore, dorsal view **C** head, frontoventral view **D** midbody leg, subventral view; **E,** both gonopods *in situ*, ventral view; **F,** right gonopod, ventral view **G, H** dissected left gonopod, subdorsal and frontal views, respectively. – Scale bars: **C, E** 0.1 mm; **D, F–H** 0.05 mm; **A, B** 0.02 mm.

In width, collum << segments 2 & 3 < head = 4 < 5 < 6=15 (16); thereafter body gradually tapering towards telson. Paraterga well-developed (Figs 30A–F, H, J), starting from a rather broadly rounded, kidney-shaped collum, mostly only faintly declivous to continue the outline of a rather slightly convex dorsum, largely set high, at about 1/3 of midbody height, with evident shoulders frontolaterally (Figs 30A–C). Caudal corner of postcollum paraterga mostly broadly rounded, obtuse-angular, more narrowly rounded and very slightly extending behind rear tergal margin only in segments 16-18 (♂) or 17-19 (♀). Lateral edge of paraterga with 2 or 3 small setigerous indentations in poreless and poriferous segments, respectively. Ozopores very evident, round, flush open on dorsal surface, clearly removed from caudal margin and lying anteriorly to bottom of caudalmost lateral incision (Figs 30B, C, E, F, K, 31B), lateral tooth being clearly shorter than medial one. Each metatergum with 3+3, long, bacilliform setae arranged in 2 or 3 regular transverse rows; polygonal bosses invisible, transverse sulcus very shallow (Figs 30A–F, K).

Legs rather long and slender, ca 1.3-1.4 (♂) or 1.1-1.2 (♀) times as long as midbody height (♂); tarsi longest and particularly slender, with modified, dense, bifid setae ventrally (Fig. 31D), but sphaerotrichomes missing.

**Figure 32.**
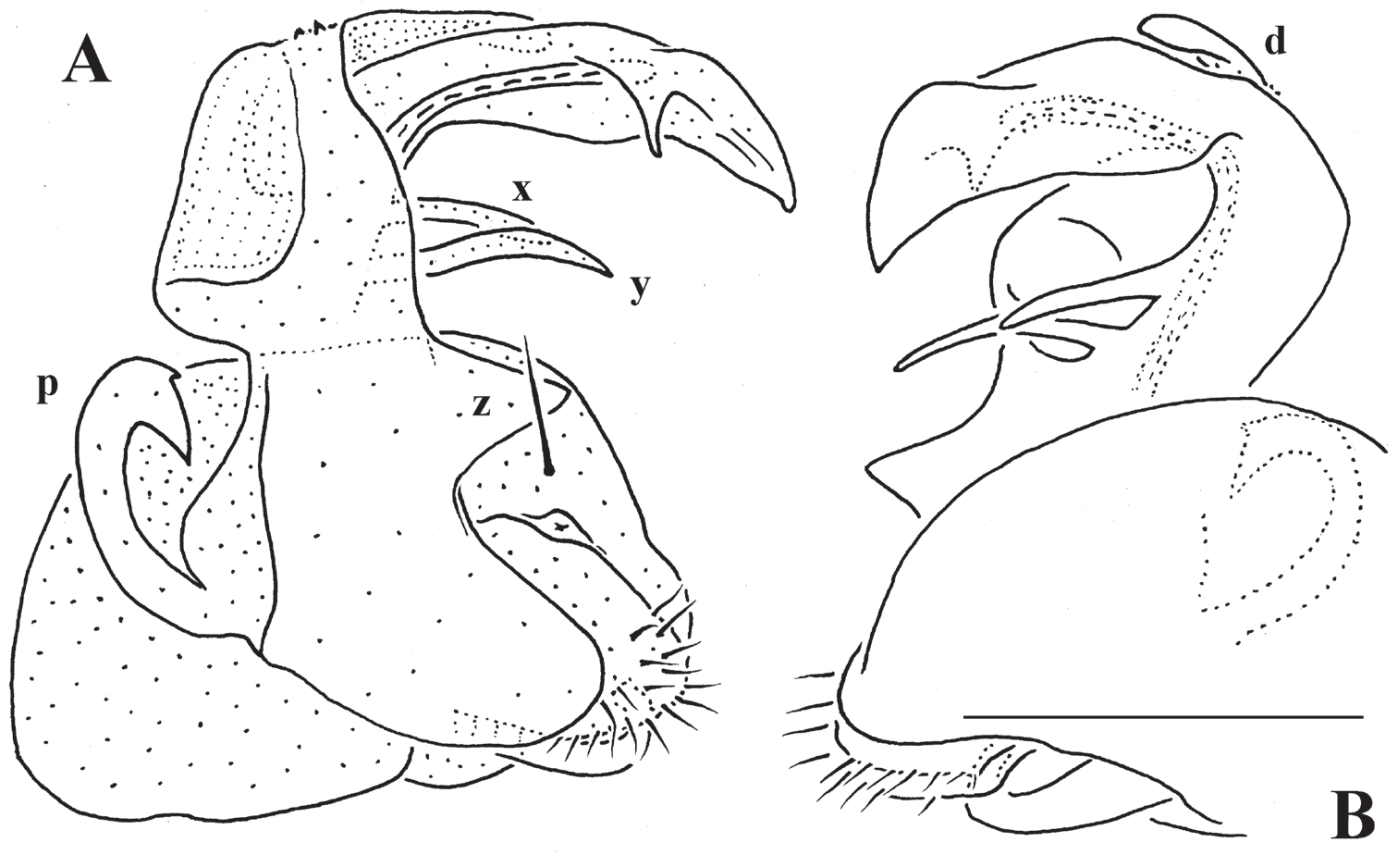
*Martensodesmus bedosae* sp. n., ♂ paratype; **A, B** left gonopod, ventral and dorsal views, respectively. – Scale bar: 0.1 mm.

Gonopod telopodite (Figs 31E–H, 32) clearly curved, but stout, unipartite; basal half especially voluminous due to an unciform frontoventral process (**p)**, more distally on caudal face with a strong subtriangular tooth (**z**) and two long spines (**x** and **y**), distal half with a short, finger-shaped, caudal process (**d**). Neither bacilliform ornamentations nor an accessory seminal chamber, nor a hairy pulvillus, seminal groove ending at base of a small subapical tooth.

##### Remarks.

This new species is still unique in showing a marked process **p** at the base of the gonotelopodite.

#### Martensodesmus spiniger sp. n.

urn:lsid:zoobank.org:act:4BB13E54-F108-46E3-A603-B45F0F67BCA1

http://species-id.net/wiki/Martensodesmus_spiniger

Figs 33 –36

##### Type material.

Holotype ♂ (IZAS), China, Guangxi, Chongzuo County, Longzhou Xian, Shanglong Xiang, Lenglei, Nonggang Forest, 106.964835°E, 22.467175°N, litter, Berlese extraction, 07.03.2005, leg. L. Deharveng & A. Bedos (CHIgx05-062).

##### Paratypes.

1 ♂ (SCAU), same locality, together with holotype; 1 ♂ (MNHN JC 345), same locality, Shanglong Xiang, Nonghang, Nonggang Forest, 106.90575°E, 22.48617°N, litter, sieving and Berlese extraction, 14.04.2010, leg. L. Deharveng & A. Bedos (CHIgx10-07).

**Non-type:** 1 ♀ (MNHN JC 345), 1 ♂ subadult, 1 ♀ (SEM), same locality (CHIgx10-07); 1 ♂ subadult (SEM), same data as holotype (CHIgx05-062).

##### Diagnosis.

Differs readily fromcongeners by the presence of only two transverse rows of setae on metaterga, combined with five strong spines on the caudal face of a rather strongly curved gonopod telopodite which lacks even traces of a solenomere.

##### Name.

To emphasize the highly spinose gonopod telopodite.

##### Description.

Length of holotype ca 4.0 mm, width of midbody pro- and metazona ca 0.35 and 0.5 mm, respectively. Length of paratype ♂ ca 4.5 mm, width of midbody pro- and metazona ca 0.4 and 0.6 mm, respectively. Length of adult ♀ ca 4.7 mm, width of midbody pro- and metazona ca 0.5 and 0.6 mm, respectively. Coloration in alcohol from uniformly pallid to head, several anterior segments and following metaterga clearly infuscate, rusty reddish, increasingly poorly pigmented towards telson.

Body with 19 (♂) or 20 (♀) segments. All characters like in *Retrodesmus cavernicola* sp. n., except as follows.

Antennae broken off, but obviously medium-sized.

**Figure 33.**
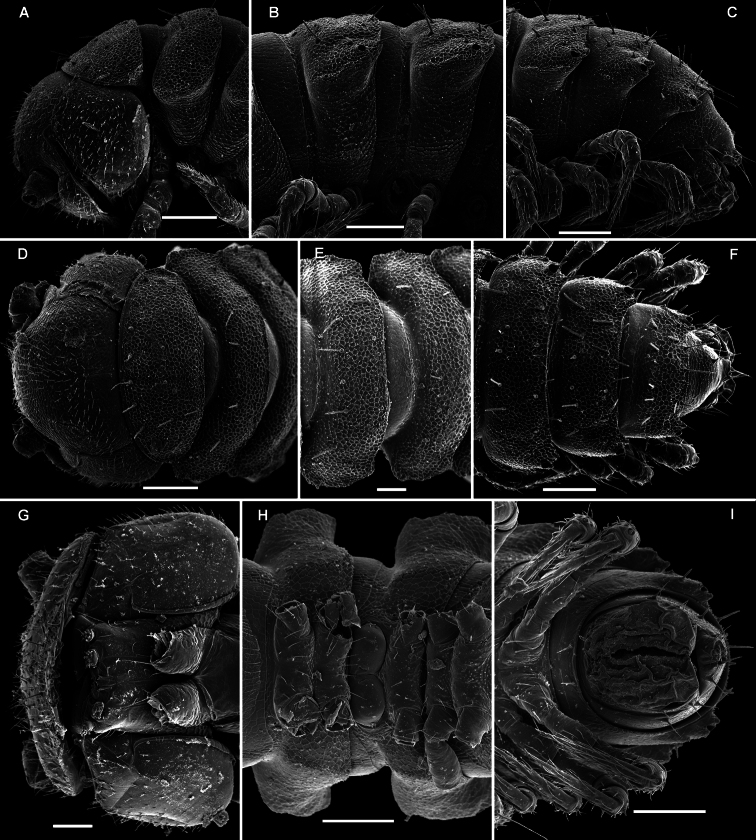
*Martensodesmus spiniger* sp. n., ♂ subadult, non-type; **A, D** anterior body part, lateral and dorsal views, respectively **B, E** midbody segments, lateral and dorsal views, respectively **C, F, I** posterior body part, lateral, dorsal and ventral views, respectively **G** head, ventral view **H** segments 6 and 7, ventral view. – Scale bars: **A–D, F, H, I** 0.1 mm; **E, G** 0.05 mm.

**Figure 34.**
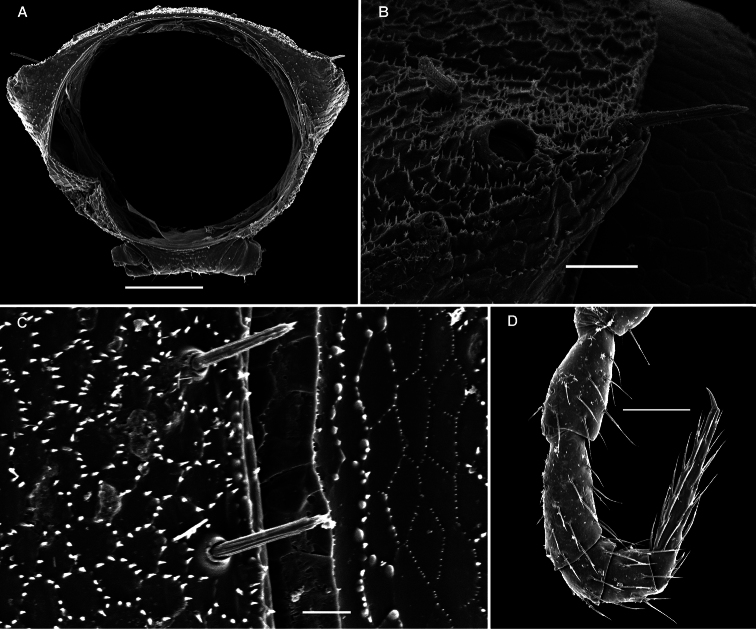
*Martensodesmus spiniger* sp. n., ♂ subadult, non-type; **A** cross-section of a midbody segment, caudal view **B** midbody paratergite with setae and an ozopore, lateral view **C** tegument texture, limbus and setae **D** midbody leg. – Scale bars: **A** 0.1 mm; **D** 0.05 mm; **B** 0.02 mm; **C** 0.01 mm.

In width, collum << segments 2 & 3 < head = 4 ≤ 5 < 6=15 (16); thereafter body gradually tapering towards telson. Paraterga medium-sized, keel-shaped (Figs 33A–F, 34A, 36C, D), a little better developed in ♂ compared to ♀, starting from a broadly rounded collum, mostly faintly declivous and continuing the outline of a rather convex dorsum, largely set rather high, at about ¼ to 1/3 of midbody height, with faint shoulders frontolaterally (Figs 33D–F, H). Caudal corner of postcollum paraterga mostly dentiform, always clearly rounded to narrowly rounded and extending increasingly well behind rear tergal margin only in segments 15-18 (♂) or 16-19 (♀). Lateral edge of paraterga with 2 or 3 small setigerous indentations in poreless and poriferous segments, respectively. Ozopores evident, round, flush open on dorsal surface, mostly clearly removed from caudal margin, lying slightly above and close to caudalmost lateral incision (Figs 33B, C, F, 34B, 36C, D), lateral tooth being shorter than medial one, in segment 17 clearly lateral, in 18^th^ caudal (Figs 36C, D). Each postcollum metatergum until 18^th^ (♂) or 19 (♀) with 3+3, long, bacilliform setae arranged in two regular transverse rows, only collum and segment 18 with three transverse rows of bacilliform setae, in segment 18 (♂) or 19 (♀) both posterior rows being placed close to each other; polygonal bosses flat, barely visible, even transverse sulcus very faint (Figs 33A–F).

Legs rather long, but stout, ca 1.4-1.5 times as long as midbody height (♂); tarsi longest and particularly slender (Fig. 34D), sphaerotrichomes or other modified setae missing.

**Figure 35.**
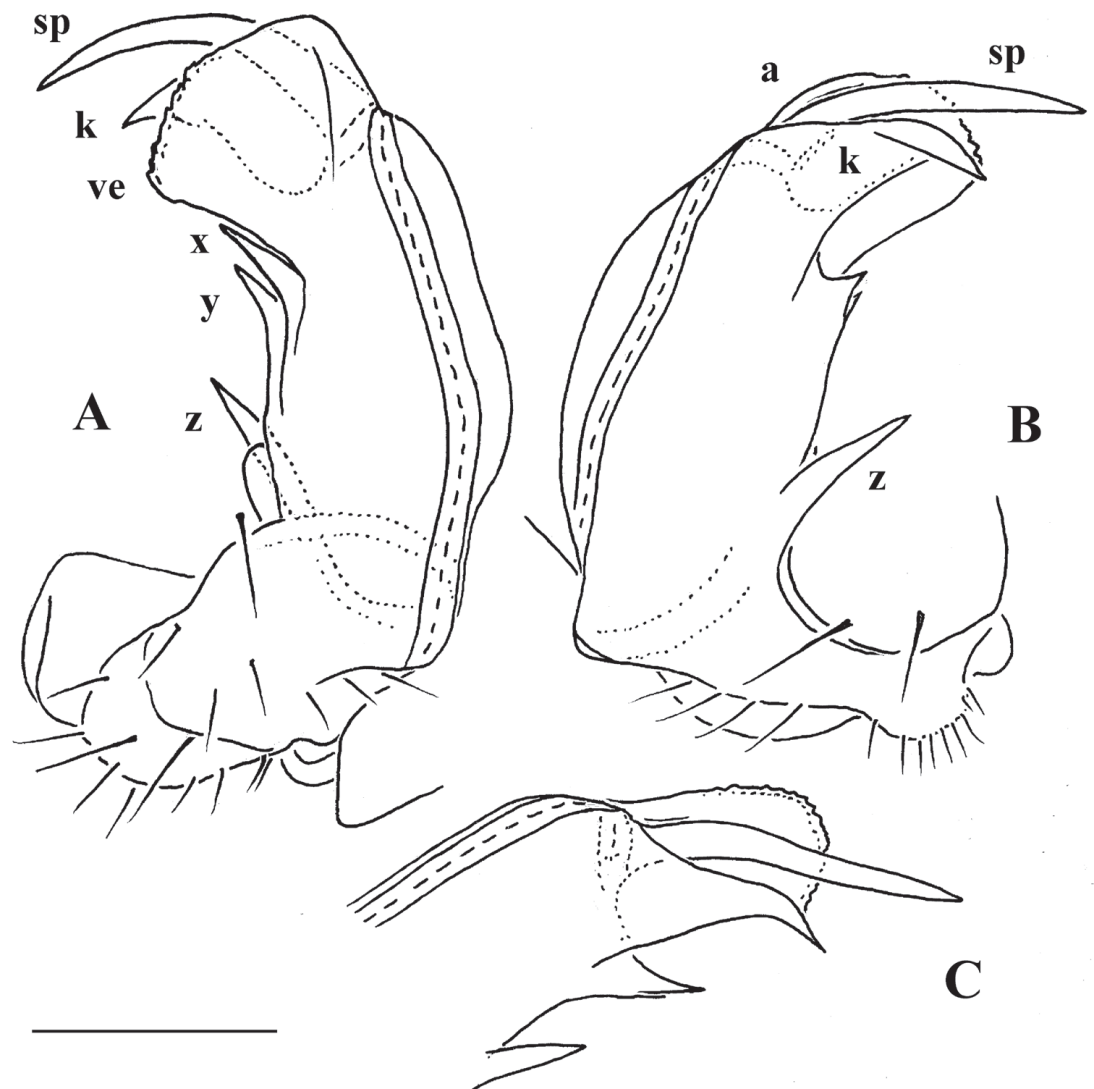
*Martensodesmus spiniger* sp. n., ♂ paratype; **A** right gonopod, ventral view **B, C** left gonopod, frontodorsal and subdorsal views, respectively. – Scale bar: 0.1 mm.

Gonopod telopodite (Fig. 35) clearly curved, but stout, unipartite; apical piece (**a**) distal to orifice of seminal groove complex, consisting of a very strong apical spine (**sp**) protected in its basal half by a membranous ventral velum (**ve**) with a faintly fringed apical margin and a similarly membranous, apically spinigerous, dorsal lobe (**k**); caudal face below **a** with three distinct spines (**x**, **y** and **z**), **z** being longest. Neither bacilliform ornamentations, nor accessory seminal chamber, nor even traces of a solenomere, nor a hairy pulvillus.

**Remarks.** This new *Martensodesmus* species appears to co-occur, even syntopically, together with another opisotretid, *Carlotretus triramus* sp. n., described just below. Moreover, these two species are superficially so similar that only adult males can be separated with confidence. We therefore prefer to regard the females and juveniles as non-type material.

#### Carlotretus triramus sp. n.

urn:lsid:zoobank.org:act:62FEDFD9-45E8-4B52-BDAD-7511C98AD4D3

http://species-id.net/wiki/Carlotretus_triramus

Figs 36 –41

##### Type material.

Holotype ♂ (IZAS), China, Guangxi, Chongzuo County, Longzhou Xian, Shanglong Xiang, Lenglei, Nonggang Forest, 106.964835°E, 22.467175°N, litter, Berlese extraction, 07.03.2005, leg. L. Deharveng & A. Bedos (CHIgx05-068).

##### Paratypes.

1 ♂ (SEM), same data as holotype (CHIgx05-066); 1 ♂ (SCAU), same locality, Shanglong Xiang, Nonghang, Nonggang Forest, 106.90575°E, 22.48617°N, litter, sieving and Berlese extraction, 14.04.2010, leg. L. Deharveng & A. Bedos (CHIgx10-07).

**Non-types.** 1 ♂ subadult (SEM), same locality, together with holotype (CHIgx05-068); 1♀ (SEM), same data as holotype (CHIgx05-064); 1♀, 3 ♀ subadults (MNHN JC 346), same locality (CHIgx10-07).

##### Diagnosis.

Differs readily from *Carlotretus setosus*, the only known congener, by the much longer, strong and totally unprotected solenomere branch, whereas the parabasal branches are slender and subunciform.

##### Name.

To emphasize the clearly triramous midlength process of the gonopod telopodite.

##### Description.

Length of holotype ca 4.3 mm, width of midbody pro- and metazona ca 0.4 and 0.55 mm, respectively. Length of paratype ♂ ca 4.6 mm, width of midbody pro- and metazona ca 0.45 and 0.6 mm, respectively. Length of adult ♀ ca 6.0 mm, width of midbody pro- and metazona ca 0.6 and 0.7 mm, respectively. Coloration in alcohol from uniformly pallid to head and metaterga faintly rusty reddish.

Body with 19 (♂) or 20 (♀) segments. All characters like in *Martensodesmus spiniger* sp. n., except as follows.

Antennae medium-sized, extending behind segment 2 when stretched dorsally.

**Figure 36.**
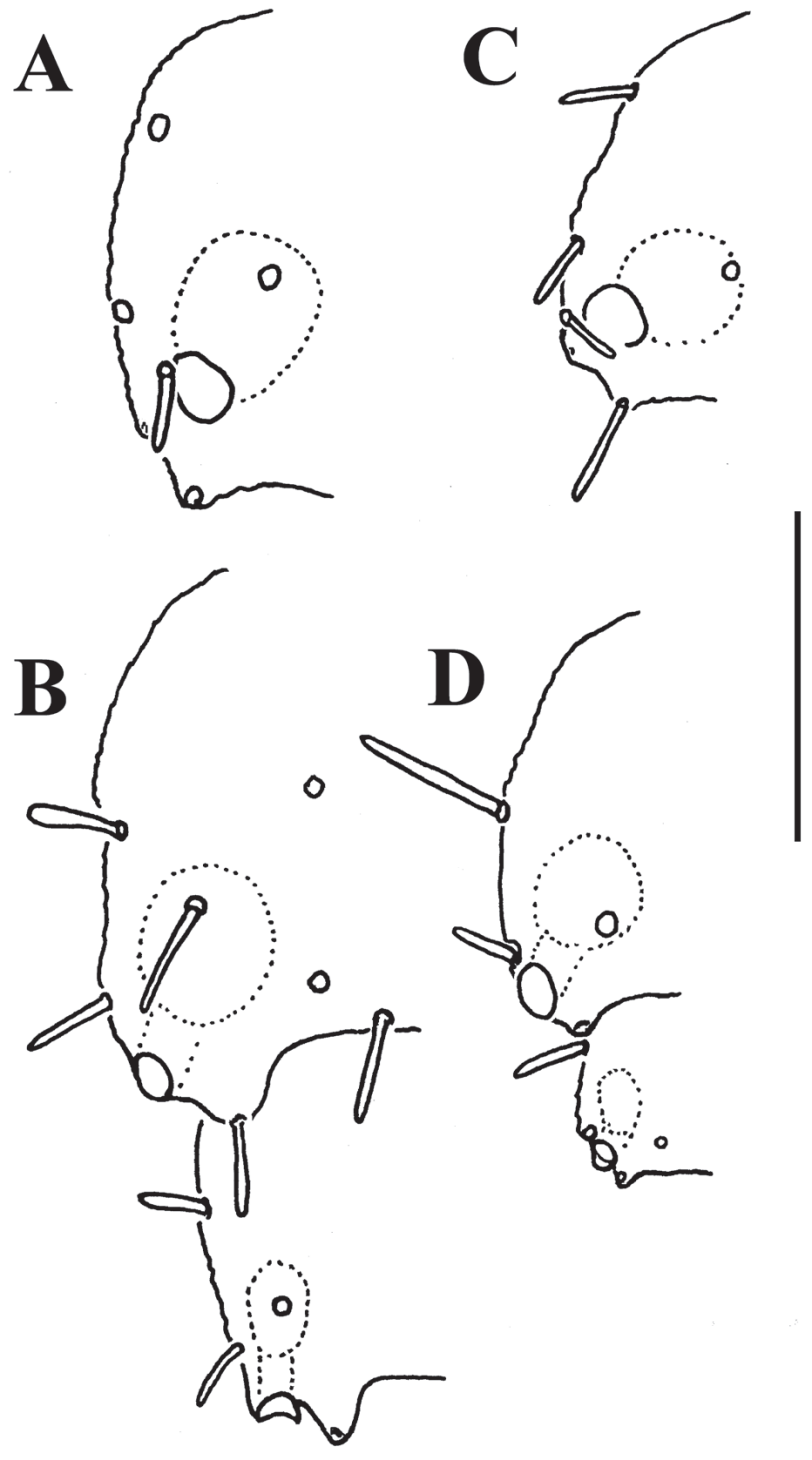
*Martensodesmus spiniger* sp. n., ♂ holotype (**A, B**), and *Carlotretus triramus* sp. n., ♂ paratype (**C, D**) **A, C** left paratergite 13, dorsal view **B, D** left paratergites 17 and 18, dorsal view. – Scale bar: 0.1 mm.

Caudal corner of postcollum paraterga mostly dentiform, always clearly rounded and extending increasingly well behind rear tergal margin only in segments 15-18 (♂) or 16-19 (♀), a little better produced behind than in *Martensodesmus spiniger* sp. n. (Figs 36A, B).

Gonopod telopodite (Figs 38B–E, 40D–H, 41) clearly curved, but its basal half quite stout, unipartite; solenomere (**sl**) very long, slender and simple, only faintly curved, orifice of seminal groove placed on a small subapical tooth, with neither bacilliform ornamentations, nor accessory seminal chamber, nor a hairy pulvillus. Two large, subunciform processes, **m** and **n**, at base of **sl**, process **n** lying more dorsally and being slightly larger than a ventral, very finely and densely microspinulate **m**.

**Figure 37.**
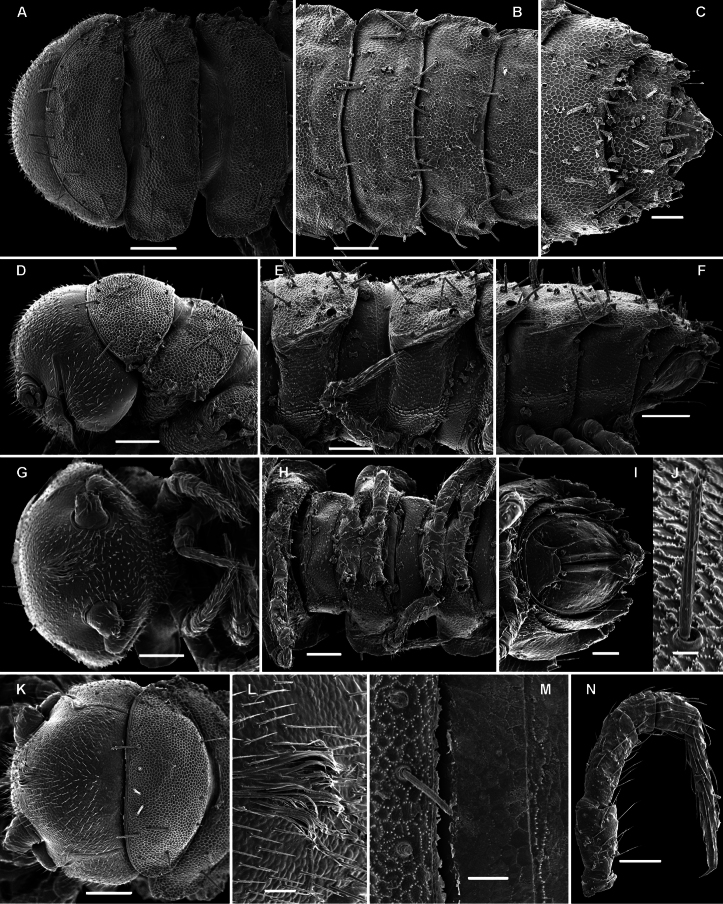
*Carlotretus triramus* sp. n., ♂ paratype (CHIgx10-07); **A, D, G, K** anterior body part, dorsal, lateral, ventral and frontodorsal views, respectively **B, E, H** midbody segments, dorsal, lateral and ventral views, respectively **C, F, I** posterior body part, dorsal, lateral and ventral views, respectively **J** tergal seta, lateral view **L, M** tegument texture, limbus and tergal seta, dorsal view **N** midbody leg. – Scale bars: **A, B, D–H, K** 0.1 mm; **C, I, N** 0.05 mm; **L, M** 0.02 mm.

**Figure 38.**
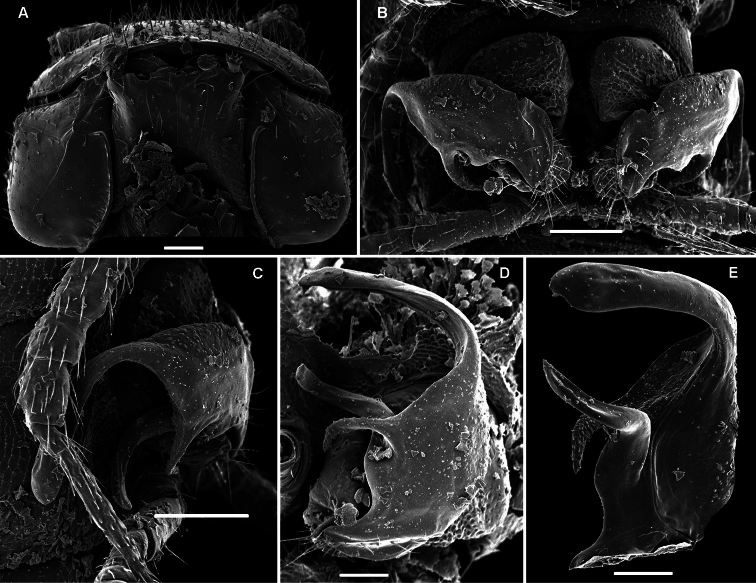
*Carlotretus triramus* sp. n., ♂ paratype (CHIgx10-07); **A** head, ventral view **B–D** gonopods *in situ* **E** dissected left gonopod, subdorsal view. – Scale bars: **B, C** 0.1 mm; **A, D, E** 0.05 mm.

**Figure 39.**
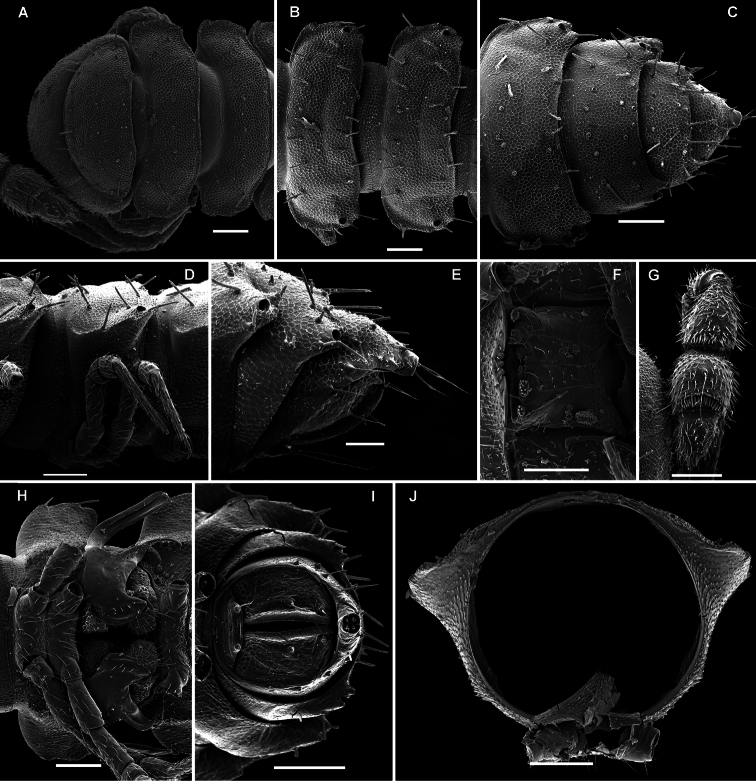
*Carlotretus triramus* sp. n., ♂ paratype (CHIgx05-068); **A** anterior body part, dorsal view **B, D** midbody segments, dorsal and lateral views, respectively **C, E, I** posterior body part, dorsal, lateral and ventrocaudal views, respectively **F** gnathochilarium, ventral view **G** antennomeres 5-8, dorsal view **H** segments 6 and 7 with gonopods *in situ*, ventral view **J** cross-section of a midbody segment, caudal view. – Scale bars: **A–D, F–J** 0.1 mm; **E** 0.05 mm.

**Figure 40.**
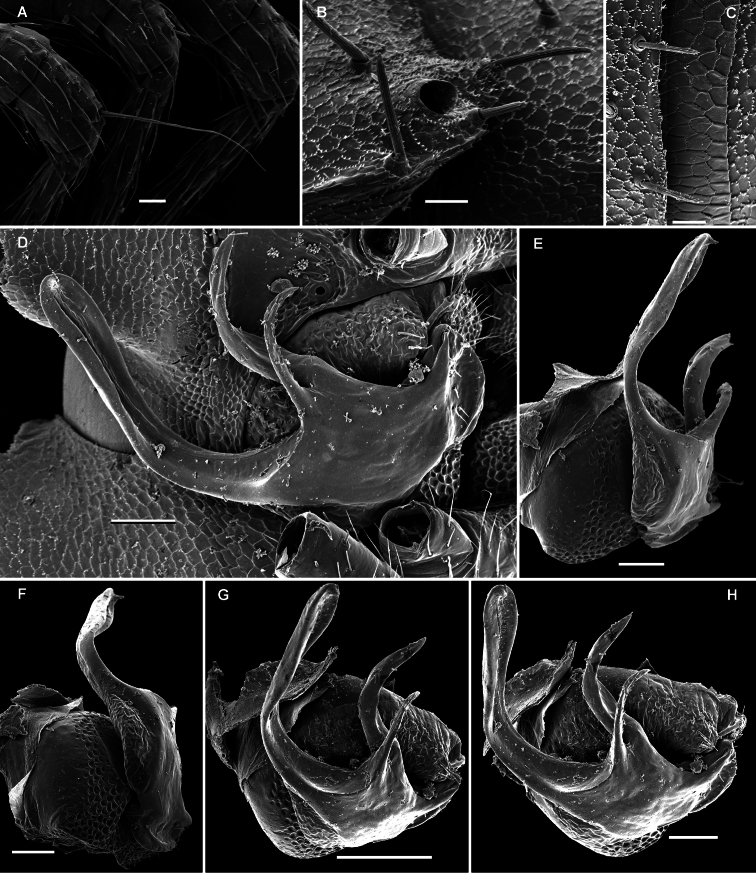
*Carlotretus triramus* sp. n., ♂ paratype (CHIgx05-068); **A** long tactile distodorsal seta on a midbody tibia **B** caudolateral corner of a midbody ozoporiferous paratergite **C** tegument texture, limbus and setae, dorsal view **D** left gonopod *in situ*, ventral view **E–H** dissected left gonopod, frontomesal, frontal, subventral and ventral views, respectively. – Scale bars: **G** 0.1 mm; **D–F, H** 0.05 mm; **A–C** 0.02 mm.

**Figure 41.**
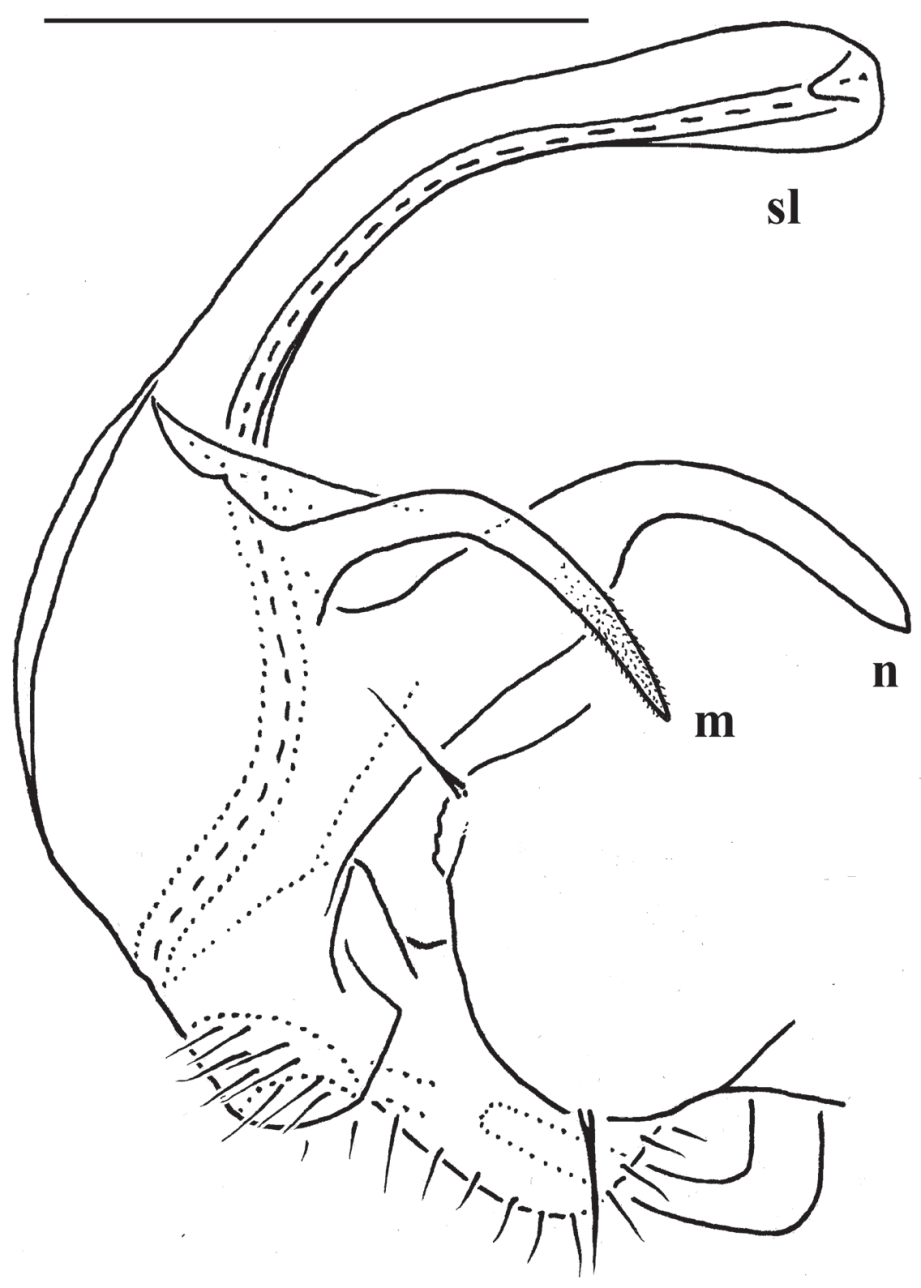
*Carlotretus triramus* sp. n., ♂ paratype (CHIgx10-07), right gonopod, mesal view. – Scale bar: 0.1 mm.

###### A key to genera of Opisotretidae, based mainly on male characters

**Table:** 

1	Adults of both sexes with 20 body segments (including telson). Prominent shoulders of paraterga causing a caudad shift of all three rows of tergal setae (Figs 11A, B). Gonopod telopodite (Fig. 11C) very simple, attenuating distad and virtually fully devoid of a trichome other than the one of a subterminal hairy pulvillus	*Opisthoporodesmus*
–	Females with 20, males with 19 body segments. Shoulders of paraterga usually not so prominent. Gonopod telopodite usually more complex	2
2	Gonopod telopodite bipartite (sl and ex), with a strong, frontobasal process p on ventral face (Figs 6, 7)	*Solaenaulus*
–	Gonopod telopodite unipartite, usually devoid of such a basal process p on ventral face	3
3	Gonopod telopodite rather stout, its distal part devoid of ornamentations (spines or setae), being a long and simple solenomere (sl), either with lobes or processes to subtend sl or with processes near sl base. Neither an accessory seminal chamber nor a hairy pulvillus (Figs 1B, C, 40D–H, 41)	*Carlotretus*
–	Gonopod telopodite variable, often with bacilliform ornamentations distally, but solenomere never so conspicuous and simple	4
4	Gonopod telopodite slender and suberect, devoid both of prominent outgrowths and distal ornamentations, at most microdenticulate near both a small accessory seminal chamber and a hairy pulvillus, with or without process p (Figs 2B, 3)	*Corypholophus*
–	Gonopod telopodite usually either clearly curved when elongate and slender or nearly straight at least in basal half when thick and stout, often with various evident outgrowths, sometimes also with bacilliform ornamentations distally	5
5	Gonopod telopodite (at least its basal half) rather stout, its basal and/or distal parts with lobes or processes, sometimes including a p; both accessory seminal chamber and hairy pulvillus wanting, but a very short, dentiform solenomere usually ornamented with a few bacilli- or setiform structures nearby often present (Figs 9, 10C, D, 29, 32, 35)	*Martensodesmus*
–	Gonopod telopodite with abundant bacilli- and/or setiform ornamentations distally	6
6	Gonopod telopodite rather stout, only slightly curved; distal part divided into a frontal stump heavily beset with bacilliform ornamentions and a simple to complex caudal branch (Figs 13B, 15C, D, 16B, C)	*Retrodesmus*
–	Gonopod telopodite slender, more clearly curved; distal part beset with ornamentations (small spines, bacilli or setae) and at least with one evident process, either devoid of or supplied with a short solenomere, but with both an evident accessory seminal chamber and a hairy pulvillus (Figs 8D, 18B–D, 20C, D, 21, 23C, D, 25, 26F, G)	*Opisotretus*

###### A key to species of *Martensodesmus*

**Table:** 

1	♂ vertex with modifications. Himalaya	2
–	♂ vertex without modifications	5
2	♂ vertex with a fossa supporting two cusps of filaments; 2+2 long bacilliform setae at the caudal edge of an elongated ♂ collum (Figs 10A, B). Gonopod as in Figs 10C, D. Bhutan	*Martensodesmus bicuspidatus*
–	♂ vertex with a hump. Gonopods different. Nepal	3
3	Gonopods as in Fig. 9	*Martensodesmus himalayensis*
–	Gonopods different	4
4	Apex of gonopod telopodite with a long slender process	*Martensodesmus sherpa*
–	Apex of gonopod telopodite with a broad, bifid, membranous lobe, devoid of any prominent processes	*Martensodesmus nagarjungicus*
5	Gonopod telopodite with a basal p, as in Figs 31E–H, 32. Guangxi, China	*Martensodesmus bedosae* sp. n.
–	Process p absent	6
6	Metaterga 2-17 (♂) or 2-18 (♀) with two transverse rows of bacilliform setae. Gonopods as in Fig. 35. Guangxi, China	*Martensodesmus spiniger* sp. n.
–	All metaterga with three transverse rows of bacilliform setae. Gonopods different	8
7	Gonopod telopodite suberect, with a broadened apex, no process proximal to apical part. Bhutan	*Martensodesmus excornis*
–	Gonopod telopodite with a strongly attenuating and curved apical half, three strong processes parabasally (Figs 28E–I, 29). Vietnam	*Martensodesmus cattienensis* sp. n.

###### A key to species of *Opisotretus*

**Table:** 

1	♂ vertex with modification	2
–	♂ vertex without modifications	3
2	♂ vertex with two paramedian tubercles (Fig. 23A). Gonopods as in Figs 23C, D. Papua New Guinea	*Opisotretus hagen* sp. n.
–	♂ vertex with a bare hump (Fig. 26A). Gonopods as in Figs 26F, G. Nusakambangan Island south off Java, Indonesia	*Opisotretus spinosus* sp. n.
3	♀ paraterga 18 and 19 with caudal teeth nearly obsolete, not extending behind rear tergal margin. Gonopod structure unknown. Java, Indonesia	*Opisotretus mimus*
–	Caudal teeth of two last paraterga at least slightly produced behind rear tergal margin. Gonopod structure known	4
4	Lateral tooth of caudalmost incision in a few last paraterga considerably longer than median one. Gonopods as in Fig. 8D. Java, Indonesia	5
–	Lateral tooth of caudalmost incision in a few last paraterga clearly shorter than median one. Gonopods different	6
5	Body about 8 mm long and 1.25 mm wide (♀)	*Opisotretus euthus*
–	Body about 12 mm long and 1.5 mm wide (♂)	*Opisotretus kraepelini*
6	Gonopods as in Figs 18B–D, 20C, D. Papua New Guinea	*Opisotretus beroni* sp. n.
–	Gonopods as in Fig. 25. Sulawesi, Indonesia	*Opisotretus deharvengi* sp. n.

## Evolutionary and biogeographic implications

The family Opisotretidae appears to range from the Ryukyu Islands, Japan and southern China, through Indochina and Indonesia, to Papua New Guinea. Simonsen (1992) dates the origin of Opisotretidae back to the Triassic (220 Ma) in relation to the geological history and fragmentation of eastern Gondwanaland, placing it mainly in the region of present-day Indonesia. However, such a reconstruction fails to account for the presence of opisotretid species all over Indonesia, including Sulawesi and New Guinea, only parts of which might have been derived from Gondwanaland. Even less convincing is the attribution of such remote and outlying areas as the Ryukyus and southern China to Gondwanaland. Thus there is no compelling reason to suppose that the evolution of Opisotretidae is linked to Gondwanaland. It seems far more logical to consider Opisotretidae as a purely Oriental family, probably the sister-group to, or even a disjunct offshoot of, the pantropical family Fuhrmannodesmidae. Opisotretids might well have originated in mainland Southeast Asia, with subsequent dispersals along the Himalayas in the West and across Indonesia (including New Guinea) in the East. Such patterns do not need to be dated as far back as the early Mesozoic, but could have instead become established much more recently, well within the Cenozoic. Moreover, Southeast Asia hosts the largest number of higher diplopod taxa, including all sixteen Recent orders of the class, and it provides many other examples, at various taxonomic levels, of connections with the Himalayas and Southeast Asian expansions to Indonesia/East Indies (Shelley & Golovatch 2011).

In the present work we place Opisotretidae in the superfamily Trichopolydesmoidea, as recently reviewed by Golovatch (2011), considering them to be close to, if not immediately derived from, the still poorly defined, highly diverse and mostly pantropical family Fuhrmannodesmidae. The reasons for this view of the relationships between them are as follows. Golovatch (1994) provided an evolutionary scenario for the genera of Fuhrmannodesmidae known from the Neotropical realm, accepting as the basalmost those genera showing rather small, subglobose gonopod coxae that form no significant gonocoel in which to hinge the largely exposed, usually rather simple and elongate telopodites. Moreover, as in some true Trichopolydesmoidea, the prefemoral (= setose) part of the gonopod is mostly orientated transversely to the body axis, extending mesally across the entire width of the coxae. Following a series of transitional states, such forms ultimately culminate in having the gonopod coxae strongly enlarged, forming a large gonocoel in which to conceal the clearly shortened, usually highly complex and deeply sunken telopodites. Their prefemoral parts already tend to be positioned increasingly parallel to the body’s main axis, thus providing a transition between the usually small-sized Trichopolydesmoidea (= so-called “micropolydesmoids”) to the normally medium- to large-sized Polydesmoidea (= so-called “macropolydesmoids”). In this respect, Fuhrmannodesmidae might well prove to be a paraphyletic group.

Naturally, similar general trends can be surmised to have occurred in the fuhrmannodesmids of the Afrotropical and, especially, Oriental realms, which support fairly diverse faunas of this family.

**Figure 42.**
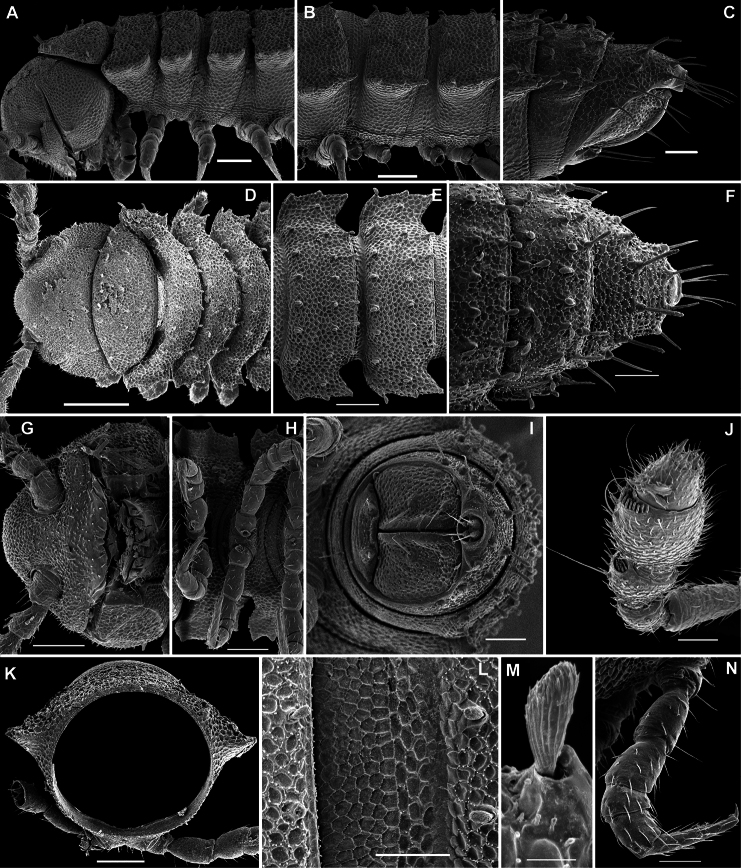
Fuhrmannodesmidae gen. sp., ♂ from near Kien Luong, Kien Giang Prov., Vietnam (Vn0308-112); **A, D** anterior body part, lateral and dorsal views, respectively **B, E, H** midbody segments, lateral, dorsal and ventral views, respectively **C, F, I** posterior body part, lateral, dorsal and ventrocaudal views, respectively **G** head, frontoventral view **J** antennomeres 3-8, subdorsal view **K** cross-section of a midbody segment, caudal view **L** tegument texture, limbus and setae, dorsal view **M** tergal seta, lateral view; **N,** midbody leg. – Scale bars: **D** 0.2 mm; **A, B, E, G, H, K** 0.1 mm; **C, F, I, J, L, N** 0.05 mm; **M** 0.005 mm.

**Figure 43.**
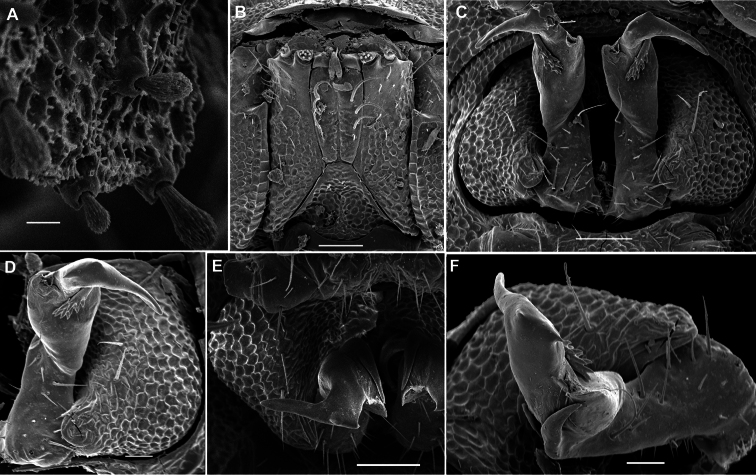
Fuhrmannodesmidae gen. sp., ♂ from near Kien Luong, Kien Giang Prov., Vietnam (Vn0308-112); **A** tegument texture and tergal setae, subdorsal view **B** gnathochilarium, ventral view **C, E** gonopods *in situ*, ventral and frontoventral views, respectively **D, F** left gonopod, subventral and sublateral views, respectively. – Scale bars: **B, C, E** 0.05 mm; **D, F** 0.02 mm; **A** 0.01 mm.

Based on the published record and the available collections of Fuhrmannodesmidae (largely kept at MNHN) from Southeast Asia and adjacent regions, including southern China, Indonesia and Melanesia (Golovatch et al., in preparation), most of the Oriental Fuhrmannodesmidae are indeed highly advanced, showing complex and strongly shortened gonopod telopodites deeply sunken inside the gonocoel formed by enlarged coxae. The orientation of the prefemoral part also varies, but it tends to be held parallel, not transversely, to the main axis of the body. However, amongst the Asian fuhrmannodesmids, there are certain genera and species that instead show quite primitive conditions, i.e. long, usually less complex gonopod telopodites that remain strongly exposed above a relatively small gonocoel. At least some of these have the prefemoral parts orientated strictly parallel to the main axis, a condition typical of the sister-superfamily Polydesmoidea. Moreover, one of these species (Figs 42, 43), from Vietnam, yet to be described, shows the distal parts of the gonopod telopodites elongated and directed laterad. This is in contrast to the much more frequent condition of the gonotelopodites in Fuhrmannodesmidae and most other groups of Polydesmida, which either cross mesally or are held parallel to each other. Based on this example, the evolution of the Opisotretidae might well be viewed as a case when the gonotelopodite of fuhrmannodesmids grows increasingly elongate and orientated laterally, while the coxae remain rather small and do not form a significant gonocoel. Against this background, we would again emphasize that none of the peripheral, non-gonopod features of Opisotretidae seems to characterize this family alone.

To summarize, the Opisotretidae could have originated directly from a disjunct member of Fuhrmannodesmidae in which the gonopod coxae would have remained small and probably been held parallel to the main axis of the body, but the development of the telopodite would have culminated in its considerable elongation and fully dorsolaterad orientation. In addition, the gonopods of this stem species must have been equipped with an accessory seminal chamber and a hairy pulvillus, the apomorphies which are absent from present-day Fuhrmannodesmidae, but retained in most of the Polydesmoidea and a few Trichopolydesmoidea, including Opisotretidae. So the ancestor of Opisotretidae might have also been a species of Polydesmoidea or even a common stem member of the Polydesmoidea+Trichopolydesmoidea. A clear-cut trend to having these apomorphies (as well as ♂ sphaerotrichomes) reduced is evident, apparently along with body miniaturization, not only in Opisotretidae, but also in some typical Polydesmidae, e.g. within the Siberian genus *Uniramidesmus* Golovatch, 1979 (Mikhaljova 2004, 2006), and Trichopolydesmidae, e.g. within the western Mediterranean genus *Cottodesmus* Verhoeff, 1936 (Verhoeff 1936, Mauriès and Vicente 1977, Mauriès 1983).

## Conclusions

There can be no doubt that many more species of Opisotretidae await discovery and description. Representatives of this family seem to be rare, but it is likely that they are seriously under-collected due to their small size. Even in caves, they seem never to be abundant, making them easy to overlook, which is in strong contrast to most diplopod groups common in tropical caves, including those of the Oriental realm (Deharveng & Bedos 2012). Sympatry, even syntopy, of two different opisotretid species also seems to be common.

## Supplementary Material

XML Treatment for Opisotretidae

XML Treatment for Carlotretus

XML Treatment for Corypholophus

XML Treatment for Martensodesmus

XML Treatment for Opisotretus

XML Treatment for Opisthoporodesmus

XML Treatment for Retrodesmus

XML Treatment for Retrodesmus dammermani

XML Treatment for Solaenaulus

XML Treatment for Retrodesmus cavernicola

XML Treatment for Opisotretus beroni

XML Treatment for Opisotretus hagen

XML Treatment for Opisotretus deharvengi

XML Treatment for Opisotretus spinosus

XML Treatment for Martensodesmus cattienensis

XML Treatment for Martensodesmus bedosae

XML Treatment for Martensodesmus spiniger

XML Treatment for Carlotretus triramus
